# Market share and recent hiring trends in anthropology faculty positions

**DOI:** 10.1371/journal.pone.0202528

**Published:** 2018-09-12

**Authors:** Robert J. Speakman, Carla S. Hadden, Matthew H. Colvin, Justin Cramb, K. C. Jones, Travis W. Jones, Isabelle Lulewicz, Katharine G. Napora, Katherine L. Reinberger, Brandon T. Ritchison, Alexandra R. Edwards, Victor D. Thompson

**Affiliations:** 1 Center for Applied Isotope Studies, University of Georgia, Athens, Georgia, United States of America; 2 Department of Anthropology, University of Georgia, Athens, Georgia, United States of America; 3 Department of Geology, University of Georgia, Athens, Georgia, United States of America; New York State Museum, UNITED STATES

## Abstract

Between 1985 and 2014, the number of US doctoral graduates in Anthropology increased from about 350 to 530 graduates per year. This rise in doctorates entering the work force along with an overall decrease in the numbers of tenure-track academic positions has resulted in highly competitive academic job market. We estimate that approximately79% of US anthropology doctorates do not obtain tenure-track positions at BA/BS, MA/MS, and PhD institutions in the US. Here, we examine where US anthropology faculty obtained their degrees and where they ultimately end up teaching as tenure-track faculty. Using data derived from the 2014–2015 *AnthroGuide* and anthropology departmental web pages, we identify and rank PhD programs in terms of numbers of graduates who have obtained tenure-track academic jobs; examine long-term and ongoing trends in the programs producing doctorates for the discipline as a whole, as well as for the subfields of archaeology, bioanthropology, and sociocultural anthropology; and discuss gender inequity in academic anthropology within the US.

## Introduction

We recently examined the realities and prospects of obtaining a faculty position in anthropological archaeology at BA/BS, MA/MS, and PhD institutions in the US and Canada [1]. Here we expand the study to include the other subdisciplines of anthropology to track and compare each of them, as well as evaluate the discipline as a whole with respect to job prospects at BA/BS, MA/MS, and PhD institutions in the US. As we outlined in our Archaeology faculty jobs study [1], repeated here to provide context for the current study, the statistics surrounding Anthropology and Archaeology career prospects provided by most outlets are not positive. In 2012, both Kiplinger Business Forecast and Forbes ranked Anthropology and Archaeology “as the worst choice of college majors with an unemployment rate of 10.5% and a median salary of $28,000 for recent college graduates” [2–3]. In their 2016–2017 forecast, Kiplinger ranked Anthropology as the fifth worst college major [4], reporting that 8,255 anthropologists and archaeologists are currently employed in the discipline. This employment estimate is only slightly higher than the 7,700 reported by the 2015 U.S. Department of Labor Outlook Handbook [5]. Kiplinger further reports that there are only approximately 920 job advertisements posted online for Anthropology and Archaeology each year. Overall, job prospects are low for the 12,000 students who successfully complete an undergraduate or graduate degree in anthropology each year. Taken together, these statistics indicate that jobs in anthropology are available for fewer than 8% of new anthropology graduates (PhD, MA/MS, and BA/BS) [4].

**Fig 1:**
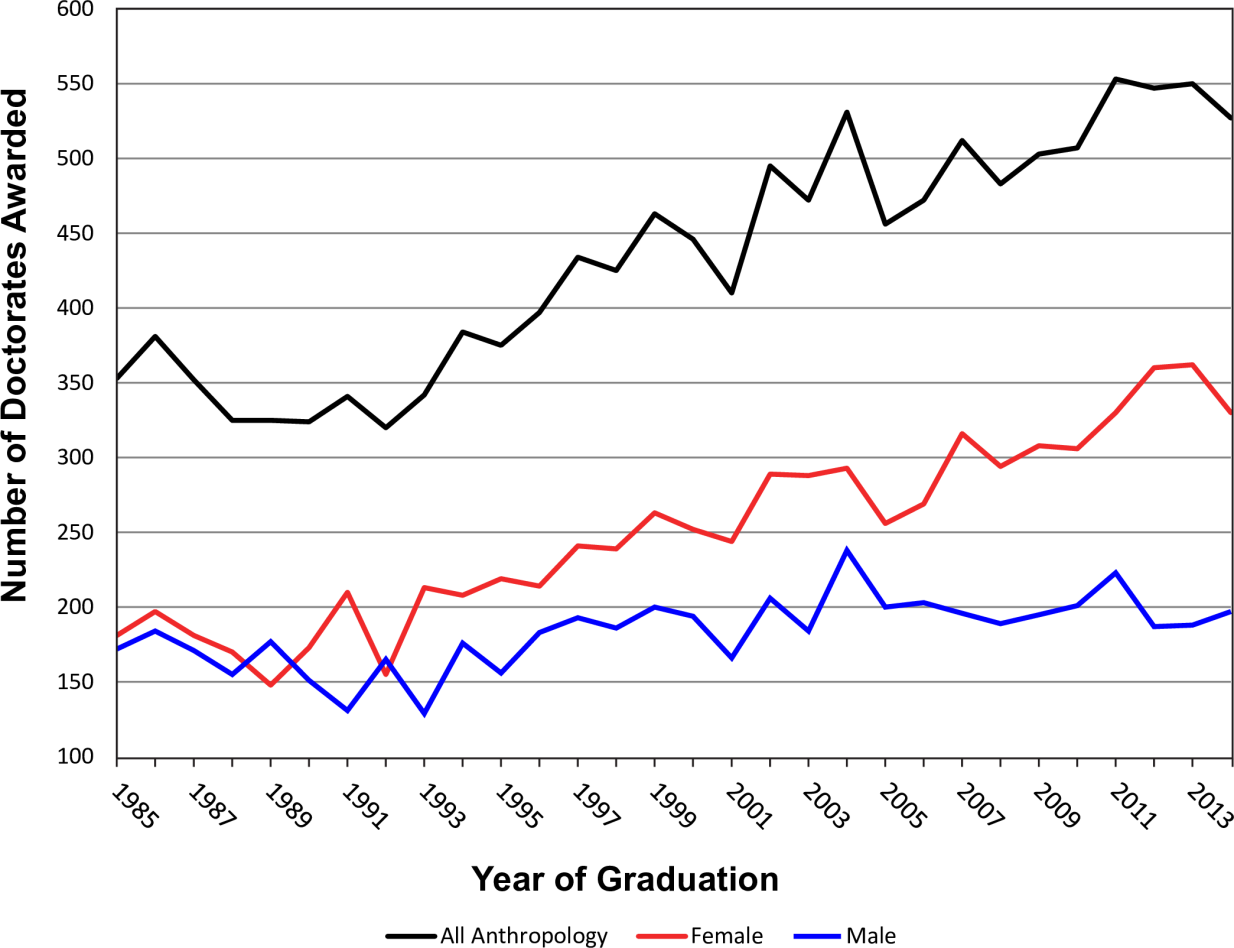
US-based PhD anthropology degrees awarded between 1985 and 2014 [6–8] showing total degrees conveyed and the gender of recipients.

The fact that most careers in anthropology require some form of advanced degree further compounds the problem. More students pursue advanced degrees in hopes of improving their career prospects within the discipline, only to find an extremely competitive, over-saturated job market for early career anthropologists. Since 1985, the number of doctoral degrees awarded by US anthropology departments has increased from approximately 350 to about 530 per year (Fig 1) [6–8]. During this same period, the number of female doctorates has increased to a point that now there are two female PhD graduates in anthropology for every male (Fig 1) [6–8]. However, an advanced degree does not necessarily lead to a better job. Those fortunate enough to hold academic positions, not only in anthropology but in other fields also, are cognizant of the fact that few of their own graduate students are likely to obtain a faculty position themselves. In today’s highly competitive academic job market, it is not uncommon to have large numbers of qualified applicants competing for a given position. Of the ca. 13,000 doctorates in anthropology conveyed in the US between 1985 and 2014 [6–8], we estimate that approximately 21% obtained tenure-track faculty positions in anthropology in the US.

A common criticism of such statistics has been that anthropology trains individuals for many sectors and career paths not captured in these numbers. While this is true, the fact remains that a large proportion of graduates who are seeking employment in anthropology will not be able to find employment within the discipline. We, of course, recognize that many programs offer non-academic pathways; however, we argue that most anthropology PhD programs are designed to train and replicate the type of scholarship engaged in by its faculty, which is by definition academic. Students are trained, largely, to be academics [9]. Thus, even when students are provided some degree of choice or training concerning different career paths, many, will choose or attempt to obtain a faculty position—this will, of course, be encouraged by faculty. In fact, encouraging students down an academic path is beneficial to faculty as it raises the overall profile of the department if their students are successful in obtaining positions. Again, we recognize this does not characterize all individuals, programs, and departments, and that there are exceptions. Despite this, however, the numbers point to the fact that there is an inordinate number of candidates—sometimes in the hundreds—for any one academic position advertised, suggesting that there are systemic issues at play.

Academic employment challenges are not limited to anthropology. Multiple studies have detailed the issues surrounding employment for the social sciences, humanities, computer sciences, and business [10–13]. In the biological sciences, the outlook is not any better. A recent study by Ghaffarzadegan and colleagues [14] determined that 84% of new PhD graduates in biological and medical sciences are not successful in landing a tenure-track academic position. Simply put, the American academic system generates doctorates at a rate greater than the job market can support [15–18].

The issue is more complex than there simply not being enough departments to hire new tenure-track faculty. The problem is that programs are producing doctorates at levels that are not sustainable. As secondary education institutions have increasingly embraced business management models over the past 30 years, universities, in order to maintain a competitive advantage, have expanded graduate programs. Many programs that used to offer a BA/BS as their highest degree now offer MA/MS degrees. Likewise, a number of programs that previously offered a MA/MS as their highest degree now offer a PhD. At the same time, many colleges and universities have moved toward limiting tenure-track faculty positions and instead use lower paid non-tenure-track faculty (NTTF) such as graduate students, part-time lecturers/instructors on short-term contracts, or full-time NTTF who typically have better job security and salaries higher than their part-time counterparts. It is estimated that NTTF comprise about 70% of the total faculty workforce as of 2011 [19–20]. Concurrent with the increase in NTTF, the numbers of full-time tenured and tenure-track faculty have decreased from approximately 55.8% in 1975 to 29.2% in 2011 [19].

Another important factor that is oftentimes overlooked with respect to the academic job market is the retirement age of tenured faculty. When the 1986 amendments to the Age Discrimination in Employment Act (ADEA) ended mandatory retirement in most occupations, postsecondary schools were allowed to continue imposing a mandatory retirement age of 70 on tenured faculty. The ADEA exemption ended in December 1993 and had an immediate effect on academic retirement rates. By 2010, the number of tenured professors older than age 65 more than doubled [21]. Currently the median age of college and university faculty surpasses all other occupational groups in the US [21]. The net result is that longer faculty careers directly translate to a reduction in the rate of new hires. Some researchers have estimated a reduction of new assistant-professor hiring rates of 10–20% [14] and have developed models that demonstrate that rather than being a transient issue, the actual number of new faculty hires has been permanently reduced [22]. In principal, for each senior-level faculty retirement, a university should be able to hire at least 1.5 tenure-track assistant professors. However, this is not necessarily the case, as many universities opt to replace tenured faculty lines with less costly NTTF hires, further suppressing the availability of new tenure-track faculty hires. The reality is that recent graduates seeking academic positions often find themselves in lengthy and insecure postdoctoral research positions [23] or a seemingly endless series of NTTF positions—if they are among the fortunate. There is little institutional incentive to replace low-paying, temporary NTTF with higher-paying tenure-track positions [23–24] and it is unlikely that this business model will change. For most recent PhD graduates, the job market—especially the academic job market—is bleak [9].

With more than 110 programs producing anthropology doctorates in the US, it is clear that not all programs are equal. Prospective graduate students must consider many factors in selecting a graduate program, including department rankings and institutional prestige, fit with departmental research interests, and, perhaps most importantly, access to financial support. Here, we examine data from US anthropology departments to: (1) identify and rank doctoral programs whose graduates have obtained tenure-track academic jobs; (2) identify long-term and ongoing market-share trends in the programs producing doctorates; and (3) evaluate gender division in academic anthropology in the US. As our primary source of data, we utilized the 2014–2015 American Anthropological Association *AnthroGuide* [25] to document the current state of the tenure-track anthropology job market in the US. Data from the *AnthroGuide* are self-reported by anthropology departments, and include general information about departments, such as degrees offered and program descriptions, as well as more specific information about their faculty. This includes academic rank, specialization, and year and origin of doctorate. Our data clearly demonstrate that a small percentage of anthropology departments hold a majority of the academic market share, which we define as the percentage of tenured/tenure-track positions in US anthropology departments that are attained by graduates of a specific program over a 20-year period of time. We then rank programs in terms of total market-share. In business, market share is determined by calculating the total sales over a given period (e.g., number of tenure-track job placements for a given period for a given program) and dividing the company’s total revenues by the industry’s total sales (e.g., total number of tenure-track anthropology jobs over a given period). Market share reflects the relative competitiveness of a company’s products or services relative to other competitors. Market share is not a measure of the real or perceived quality of goods and services, nor is it a measure of how many individual units a company placed on the market, but is instead a measure of how company sales compare to other companies within the same industry. Likewise, our academic market share rankings are not a measure of the real or perceived quality of a graduate program, nor are they a reflection of how many PhD graduates from a given program entered the tenure-track job market—market share is simply a measure of tenure-track job placement for individuals from a specific university relative to other universities.

The idea of ranking programs based on placement is not a new concept. Schmidt and Chingos [26] proposed the idea that a graduate program’s history of placing new scholars into faculty positions is as important of an indicator of departmental quality as the research produced within it. As part of their study, Schmidt and Chingos [26] compared academic job placement for recent political science doctorates to National Research Council (NRC) Q ranking scores. They determined that their per-capita academic placement rankings were fairly strongly correlated (R = 0.871) with the NRC rankings, but that in some cases lower-ranked NRC programs had better per-capita academic job placement than some of the higher ranked NRC programs. The authors concluded that ranking programs using the concept of quality is difficult to measure precisely, but that a program’s placement record reflects both the quality of the students it is able to attract as well as the training they receive.

More recently, Speakman and colleagues [1] examined academic tenure-track job placement at BA/BS, MA/MS, and PhD institutions for programs producing anthropological archaeology doctorates in the US and Canada. This study determined that a small number of graduate programs in the US and Canada account for a majority of the total market-share. This work also concluded that for prospective doctoral students, obtaining a degree from the right program could be among the most critical first steps for those who aspire for a tenure-track academic career. In the current paper, we examine market share for anthropology as a discipline. Unlike the Speakman et al. study, which focused on the archaeology and bioarchaeology job market in the US and Canada [1], here we limit our geographical focus to doctoral programs in the US and expand our discussion to include the subdisciplines of sociocultural anthropology and biological anthropology, as well as archaeology.

At this point, we feel the need to emphasize several key concepts that are stated in detail in our previous study [1]. First, our program rankings describe only market share in terms of total numbers of graduates who have obtained tenure-track positions in BA/BS, MA/MS, and PhD programs within the US. Our rankings do not reflect the real or perceived quality of a graduate program (although arguably these variables are highly correlated). We do not attempt to, nor can we account for, PhDs working outside of academia in the US (e.g., industry, government, museum, NGO’s, etc.) or anthropology PhDs who are faculty in non-anthropology departments or PhDs who have found academic employment in countries other than the US. Our focus here is solely on tenure-track faculty in anthropology departments in the US who are employed in BA/BS, MA/MS, and PhD programs. Our database only includes individuals listed as “faculty”. Faculty generally hold the title of “assistant,” “associate,” or “full” professor. Information for people classified as anything other than “faculty” is not included in our database. These include categories such as lecturer, staff, post-doc, research staff, adjunct, affiliated, emeritus, museum professional, or anthropologists in other departments. We do not assume that all graduate students entering a PhD program begin with the goal of obtaining a tenure-track position, but arguably many incoming PhD graduate students entertain the idea of completing their degree and entering the tenure-track job market. Finally, our focus on tenure-track employment in BA/BS, MA/MS, and PhD programs is not meant to imply that other career tracks are more or less satisfactory and/or fulfilling—our focus here is simply on the tenure-track job market.

## Results and discussion

As a first step in examining the data, we compiled a Table (S1 Table) in which we tabulated total market share for each of the approximately 118 US universities (as well as dozens of foreign institutions) that have (or previously had) anthropology PhD programs. We only include in this Table programs that placed at least one individual who graduated between 1994 and 2014 into an anthropology faculty position within the US. We then tabulated PhDs completed between 1994 and 2014 (n = 2070) and sorted the 20-year total from highest to lowest. Because some programs tend to emphasize preferentially archaeology, bioanthropology, or sociocultural anthropology, we also produced comparable Tables for each of these subdisciplines (S2–S4 Tables). In an ideal study, we would we able to compare total numbers of graduates per institution directly to numbers of graduates who obtained faculty positions to provide indices of per-capita tenure-track placement for graduates of a given institution—however, these data simply do not exist.

We calculated percentiles to rank each university based on their cumulative 20-year market share. We used cutoffs of 95^th^, 90^th^, 75^th^, 50^th^, 25^th^, and 10^th^ percentiles to rank the programs. These rankings are summarized in Table 1. Comparable Tables for the subdisciplines of archaeology, bioanthropology, and sociocultural anthropology are presented in S5 Table. Programs that had no anthropology faculty job placements in the past 20 years (ca. 10) are not included in our assessment. It is important to note that many, but not all, of the lower-ranked programs are relatively new (e.g., past 20 years) and that the low rankings for these programs are not as informative as rankings for older and more established programs.

**Table 1:** Summary statistics for ranking and placement averages for anthropology programs (1994–2014). Summary statistics for ranking and placement averages for Anthropology programs listed in S1 Table.

Percentile	Institutions	Placements (1994–2014)	Market Share % (1994–2014)			20-year placement average per institution	Average placement per institution per year	Average years per placement
95th	6	567	27.4			94.5 ± 20.9	4.73	0.2
90th	6	244	11.8			48.8 ± 3.1	2.03	0.4
75th	19	604	29.2			31.8 ± 6	1.59	0.6
50th	27	447	21.6			16.6 ± 3.8	0.83	1.2
25th	25	164	7.9			6.6 ± 2.3	0.33	3.0
10th	28	44	2.1			1.6 ± 0.8	0.08	12.7

For the discipline of anthropology, there are 5 programs in the 95^th^ percentile. The 95^th^ percentile also includes all foreign-derived doctorates grouped as a single category “Foreign.” There are 6 departments in the 90^th^ percentile. The 11 universities and foreign-derived doctorates in the 90^th^ and 95^th^ percentiles account for more than 39% of the total market share over the past 20 years. Departments in the 95^th^ percentile average 4.7 graduates landing tenure-track positions per year, and programs in the 90^th^ percentile average slightly more than 2 placements per year (Table 1, S1 Table). There are 19 universities in the 75^th^ percentile, with an average placement of 1.6 graduates per year. When we consider departments ranked at the 75^th^ percentile or higher, 30 PhD programs (ca. 27% of all US doctoral programs) and foreign-derived degrees account for more than 68% of the total market share.

The 27 programs in the 50^th^ percentile, 25 in the 25^th^ percentile, and 28 in the 10^th^ percentile are placing doctorates on average at a rate of one individual every 1.2 years (50^th^ percentile), one every 3 years (25^th^ percentile), or one person every 12.7 years (10^th^ percentile). We note that a few universities in the 10^th^ percentile do not actually have doctoral programs in anthropology. These programs/individuals represent faculty who obtained doctorates outside the field of anthropology and gained employment within an anthropology department.

Considering that programs ranked lower than the 75^th^ percentile account for 80 universities (or 72% of all doctoral programs), yet have only 31.6% of the total market share, it is critical that potential graduate students understand that although there are many choices available for graduate school. The harsh reality is that not applying and/or being accepted to a top school has serious ramifications if one has aspirations of obtaining a tenure-track academic position. We likewise point out that departments have different strengths in archaeology, bioanthropology, and sociocultural anthropology and that just because a program is ranked highly for all subdisciplines of anthropology (Table 1, S1 Table) that one must consider the job placement rates for program within their specific subdiscipline (S2–S5 Tables). In Table 2A–2D, we list all programs in the 90^th^ percentile or higher for each subdiscipline of anthropology. The University of Michigan, Harvard University, and foreign earned doctorates are ranked highly in all three subdisciplines. We note, however, that data for Harvard subsume two separate doctoral programs: anthropology and human evolutionary biology (i.e., biological anthropology).

**Table 2:** List of programs in the 90th percentile or higher. All subfields of anthropology (A), archaeology (B), bioanthropology (C), and sociocultural anthropology (D). The number of placements (n) and percentage of academic market share (%) for the period 2004–2014 are provided.

**A** *Anthropology (All Subdisciplines)*			
University	n	%	Percentile
Univ. Chicago	115	5.6	95th
Foreign	114	5.5	95th
Univ. Michigan	106	5.1	95th
Univ. California, Berkeley	90	4.3	95th
Harvard Univ.	79	3.8	95th
Univ. Arizona	63	3	95th
Univ. Texas, Austin	52	2.5	90th
Univ. Pennsylvania	51	2.5	90th
Univ. California, Los Angeles	49	2.4	90th
New York Univ.	48	2.3	90th
Yale Univ.	44	2.1	90th
Stanford Univ.	41	2	90th
**B** *Archaeology*			
University	n	%	Percentile
Univ. Michigan	31	6.5	95th
Foreign	30	6.3	95th
Univ. California, Berkeley	25	5.2	95th
Univ. Arizona	24	5	95th
Harvard Univ.	18	3.8	90th
Arizona St. Univ.	17	3.5	90th
Univ. Pennsylvania	16	3.3	90th
Texas A&M Univ.	15	3.1	90th
**C** *Biological Anthropology*			
University	n	%	Percentile
Foreign	23	5.3	95th
SUNY Stony Brook	20	4.6	95th
Harvard Univ.	19	4.4	95th
Emory Univ.	18	4.1	95th
Univ. Michigan	17	3.9	90th
Univ. New Mexico	15	3.4	90th
Univ. Tennessee	14	3.2	90th
Ohio St. Univ.	14	3.2	90th
**D** *Sociocultural Anthropology*			
University	n	%	Percentile
Univ. Chicago	95	8.2	95th
Foreign	61	5.3	95th
Univ. Michigan	58	5	95th
Univ. California, Berkeley	55	4.8	95th
Harvard Univ.	42	3.6	95th
New York Univ.	38	3.3	90th
Univ. Arizona	34	2.9	90th
Univ. Texas, Austin	33	2.9	90th
Columbia Univ.	31	2.7	90th
Stanford Univ.	31	2.7	90th

**Fig 2:**
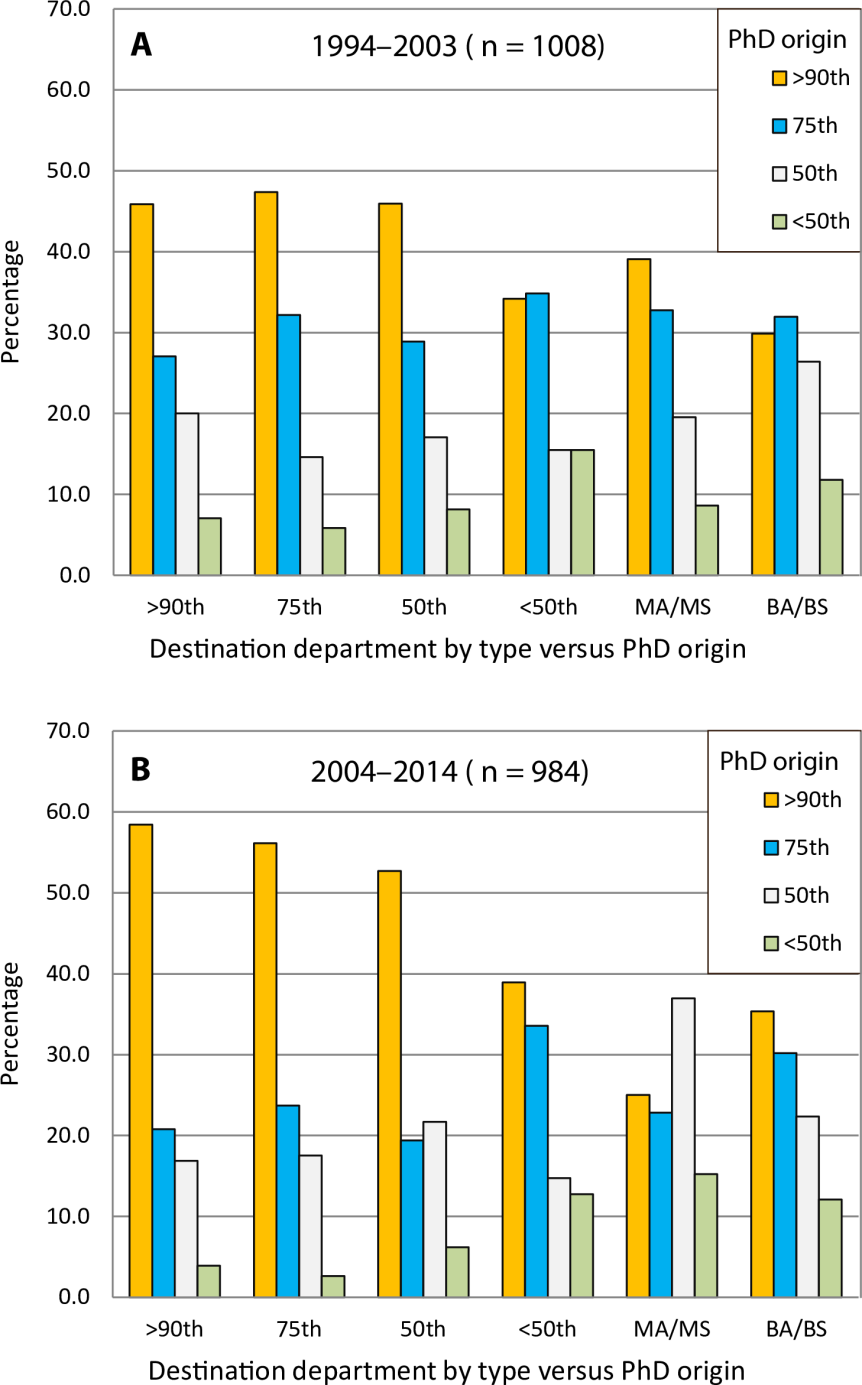
**Comparison of PhD origin versus the destination department (where the individual is ultimately employed) for 1994–2003 (A) and 2004–2014 (B).** The x-axis depicts the type of program that PhD graduates are hired into relative to the rank of the program from where individual graduated. Academic market share change over the past 20 years has increasingly favored the hiring of PhD graduates from anthropology programs in the 90^th^ percentile and higher into higher ranked anthropology PhD programs.

We examined two groups of PhD recipients: those who graduated between 1994 and 2003 (n = 1008) and those who graduated between 2004 and 2014 (n = 984). Using the percentile categories defined above—which again describe market share, not the real or perceived quality of a graduate program—we plotted where anthropologists originated, and where they ultimately obtained faculty positions (Fig 2). It is evident from the two periods examined that where an individual obtains their PhD greatly influences where they ultimately become employed. It is not surprising that graduates from programs ranked in the 75^th^ percentile and higher dominate the job market for Period 1994–2003 with the exception of departments that offer a BA/BS as their highest degree. Interestingly, for the period 2004–2014, graduates of programs ranked at the 90^th^ percentile and higher are being hired at higher rates than previously into departments with doctoral programs that are ranked in the 50^th^ percentile and higher. Although we have no means by which to evaluate individuals who obtained positions in higher ranked departments, it appears that those who obtain doctorates from foreign institutions or higher-ranked programs (≥90^th^ percentile) currently have a much greater competitive advantage in the academic job market than in the previous period. In contrast, individuals who earned doctorates from programs ranked below the 50^th^ percentile are less likely to obtain positions in higher ranked PhD programs and are more likely to obtain positions in lower-ranked PhD departments and departments that offer a BA/BS or MA/MS as their highest degree.

**Fig 3:**
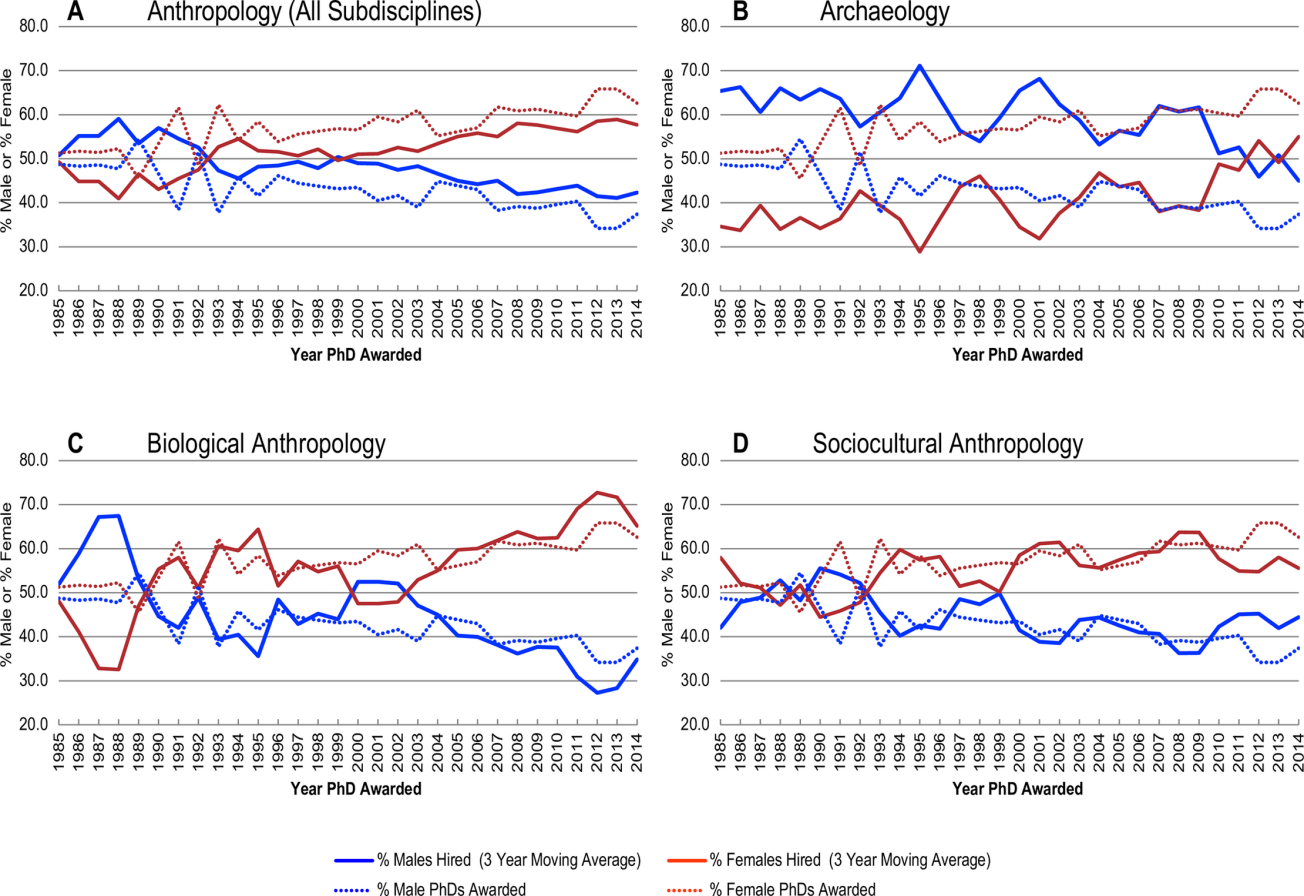
Percentages of male and female anthropology doctorates awarded by year versus percentages of males and females hired into tenure-track anthropology department positions. Assuming proportional distribution in hiring and comparable enrollment/completion of males and females among the subfields, solid and dashed lines of the same color should agree. For the discipline as a whole (A), males have been more successful over the past 30 years in obtaining tenure-track jobs relative to the actual percentage of male PhD graduates. From approximately 1992–2009, the subfields of sociocultural (D) and biological anthropology (C) were successful in hiring males and females at rates that reflect the actual percentages of male and female PhD graduates whereas males in archaeology (B) have been systematically hired at disproportionate rates relative to percentages of female graduates. Of note is that disparity in hiring has increased in sociocultural (D) and biological anthropology (C) following the end of the recession in 2009, whereas hiring disparity has decreased in archaeology (B) since 2009.

Finally, we examined differences in gender in hiring practices for anthropology as a whole and the subdisciplines of archaeology, biological anthropology, and sociocultural anthropology. Using NSF data [6–8], we modeled the percentages of male and female anthropology doctoral recipients from 1985 to 2014 (S6 Table; Fig 3A, dashed lines). We note that a well-balanced department does not necessarily mean that there are equal numbers of male and female faculty. Instead, we argue that current hiring rates should reflect the graduation rates for males and females. Based on graduation rates for males and females over the past three decades, we suggest that a well-balanced department should be hiring more females than males. This is especially true given that since 1993, 60% of all anthropology doctoral recipients are female (S6 Table). To examine disparity in hiring practices, we projected the percentages of males and females hired into tenure-track anthropology positions (S6 Table; Fig 3A, solid lines) by their year of graduation using a 3-year moving average. The model shows that although gender equity has greatly improved in recent decades, especially since 1999, males continue to be disproportionately hired into tenure-track positions. We applied the same proportional distribution model to the subfields of archaeology, biological anthropology, and sociocultural anthropology (Fig 3B–3D) and projected the percentages of males and females hired within each subdiscipline. Implicit in this model is the idea that the relative percentages of males and females in anthropology are more or less constant across the subfields. Based on our knowledge of the discipline, we believe this to be accurate. Several trends are observed. First, from approximately 1992–2009, the subfields of sociocultural and biological anthropology have been most successful in terms of hiring males and females at rates that reflect the actual percentages of males and females who earned doctorates. In contrast, during the same period, hiring practices in archaeology are strongly biased toward males. Second, since approximately 2009, disparity in hiring has increased. Specifically, males are being hired into sociocultural anthropology positions—which represents about 55% of all anthropology faculty hires—at disproportionately higher rates than females. In biological anthropology, similar trends are observed. Third, since 2009, the number of male archaeologists hired into academic positions has decreased dramatically. Although disparity still exists, more female archaeologists are being hired than previously.

**Fig 4:**
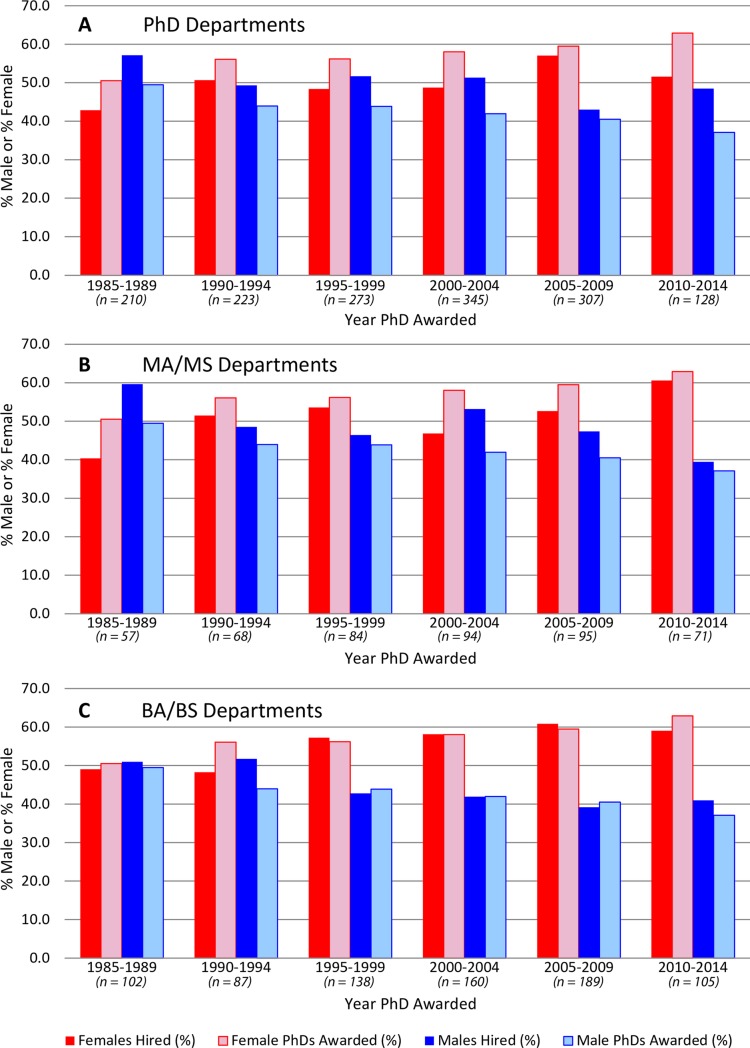
Observed versus expected male and female hiring trends for anthropology departments divided into 5-year increments based on the year an individual graduated and the department type (highest degree offered) an individual is hired into. (A) PhD programs, (B) MA/MS programs, and (C) and BA/BS programs. Males have been systematically over hired into PhD departments (A) and MA/MS For BA/BS departments (C), the percentages of females hired have been more or less equal to the percentage of female PhD graduates with the exception of the period 1990–1994.

Whereas it is informative to understand overall hiring trends of male and female anthropologists, it also of interest to examine the types of anthropology departments into which males and females are being hired. We therefore examined the percentages of males and females hired into three types of anthropology departments: (1) departments that offer a PhD as their highest degree, (2) departments that offer a MA/MS as their highest degree, and (3) departments that offer a BA/BS as their highest degree. We modeled actual hiring rates (based on year of graduation) against NSF graduation rates [6–8] and looked at each type of department in 5-year increments for the period 1985–2014 (Fig 4). The idea here is that assuming all things are equal, males and females should be hired at percentages that reflect graduation rates. For example, if we look at BA/BS departments (Fig 4C), we observe that for most of our 5-year increments that males and females are being hired at rates that are proportional to actual graduation rates. In contrast, males are consistently being hired into PhD-granting departments at rates higher than what our proportional distribution model suggests is equitable, while females are being under-hired. For PhD-granting departments (Fig 4A), equity in hiring was almost achieved during the period 2005–2009, but during the period 2010–2014, it appears that males are again being over-hired by PhD granting departments. In fact, hiring disparity for this particular group exceeds all other categories since 1985. Additionally, we observe that males have been consistently over-hired into MA/MS departments for the past 30 years (Fig 4B).

**Fig 5:**
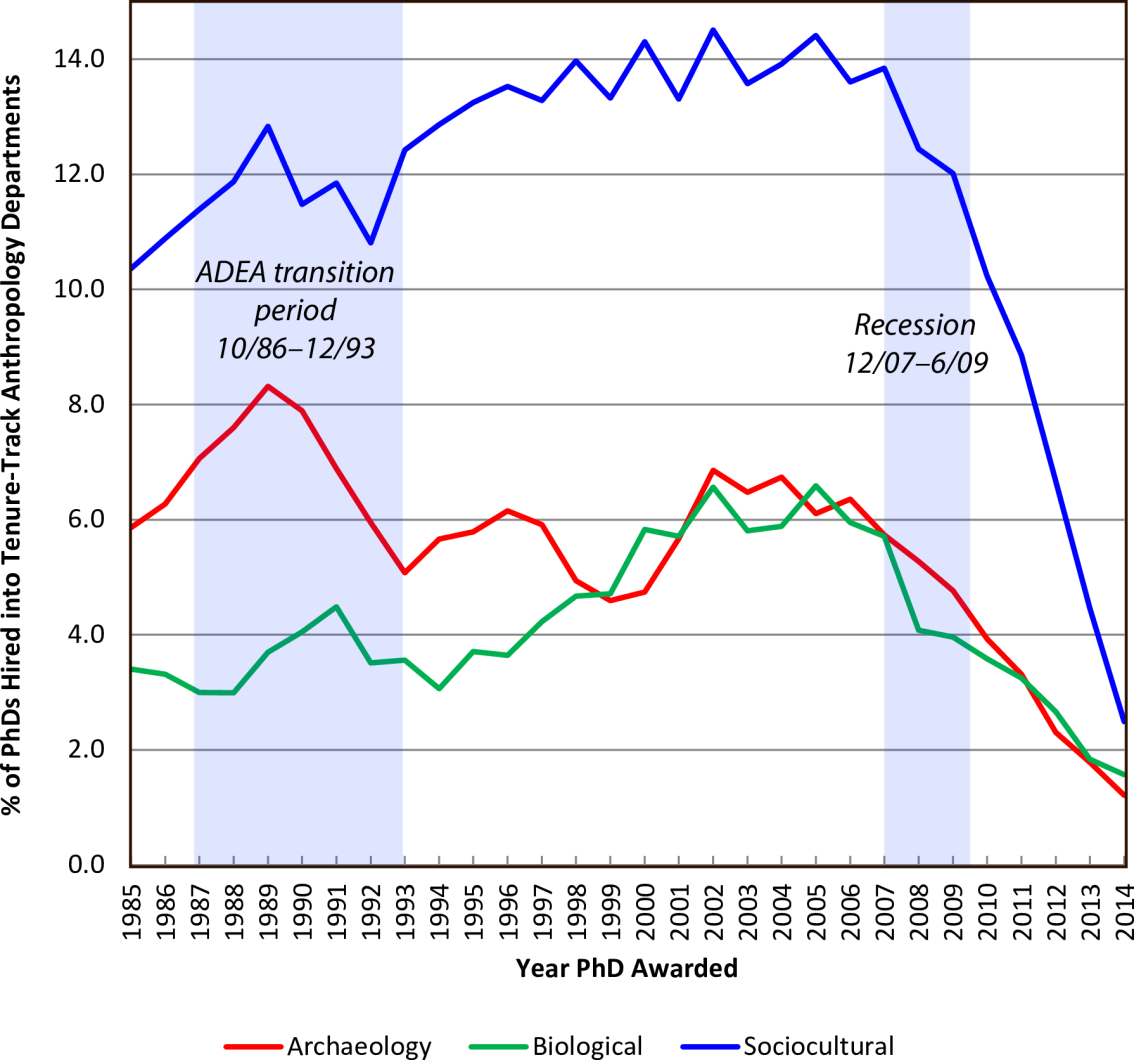
Hiring trends for PhD anthropology doctoral graduates by subfield (archaeology, biological, and sociocultural) from 1985–2014. The 2007–2009 Recession and the transition period for the 1986 Age Discrimination Employment Act (ADEA) which ended mandatory retirements of tenured faculty effective December 1993 are highlighted. Data for year of graduation are smoothed using a 3-year moving average.

As a final exercise, we examined the percentages of PhDs, by subdiscipline, who have obtained tenure-track positions since 1985 (Fig 5). When we plotted these percentages by year, two trends were apparent. The first trend is related to the ADEA exemption ending mandatory faculty retirements in December 1993. Concurrent with the end of the ADEA exemption, we observe a decrease in faculty positions for all subfields. The second trend directly relates to the financial crisis of 2007–2009 which ultimately led to the worst global recession since the 1930s. At the onset of the recession, academic institutions were forced to enact cost-saving measures, including an increased reliance on NTTF positions. Additionally, some faculty who would have retired or who were approaching retirement chose to delay retirement because the value of their retirement portfolios had been reduced or decimated. As Kaskie [21] has shown, the median age of faculty now surpasses all other occupational groups in the US. Using the 1982 median age of 30.8 for social scientists at the time of their doctorate [27], we estimate during the 2014–2015 academic year that approximately 6% of all anthropology faculty were age 70 or higher and that about 14.5% of all anthropology faculty were age 65 or older. The cumulative effect of the recession, increased use of NTTF by higher education institutions, and more anthropology doctorates entering the workforce is readily apparent in Fig 5 (see also S6 Table). Recent doctoral graduates are simply not successful in obtaining tenure-track faculty jobs at rates anywhere similar to those observed prior to the onset of the 2007 recession.

Our current broader study comes to the same conclusion as our earlier one that focused specifically on archaeology [1]. That is, obtaining a tenure-track faculty position in anthropology, no matter the subdiscipline, is exceedingly difficult and the harsh reality is that programs across the US produce far more PhDs than can be sustained in the tenure-track job market. As shown in Fig 1, there has been a steady increase in the numbers of Anthropology PhDs over the past 30 years and recent graduates are increasingly not successful in obtaining faculty positions (Fig 5, S6 Table). This situation is compounded by a number of factors that includes changes to mandatory retirement ages, the recent recession, expansion of graduate programs, and an increase in the use of NTTF by colleges and universities.

Based on NSF data [6–8], we know that between 1995 and 2014 a total of 9,558 Anthropology doctorates (all subfields) were conferred in the US. According to our database, derived from the 2014–2015 *AnthroGuide* [25], approximately 1,989 individuals who graduated between 1995 and 2014 from a US institution were employed as tenure-track anthropology faculty at BA/BS, MA/MS, and PhD institutions in the US. These data indicate a faculty employment rate of approximately 21% for those who graduated since 1995. We acknowledge that some programs focus on PhD training for foreign nationals from Latin America, Africa, and/or Asia who then return to their country of origin to pursue academic positions. Nonetheless, it is apparent that only about 1 in 5 US-derived anthropological PhDs is successful in getting a tenure-track faculty position in a US anthropology department. To compound the situation even further, US doctorates face competition from individuals (both US citizens and foreign nationals) who earned doctorates outside the US and in some cases from individuals who earned doctorates from humanities, geoscience, and/or biology programs. As we observed in Table 1, a total of 5.5% of the market share for individuals who graduated between 1994 and 2014 earned their doctorate in countries outside the US.

The 2007–2009 recession had a profound impact on the numbers of new tenure-track positions in anthropology. A total of 591 individuals who graduated between 2004 and 2009 obtained faculty positions at BA/BS, MA/MS, and PhD institutions in the US. In contrast, only 304 individuals who completed doctorates between 2010 and 2014 obtained tenure-track jobs. We recognize there is a lag of a few years for most individuals from the time they complete their doctorate until the time they obtain a faculty position; however, we do not expect placement rates to return to pre-recession numbers anytime soon. This situation results in a compounding number of applicants every year. If there are 500 PhD anthropologists who graduate every year for 5 years who are intent on getting a faculty position, and there are only 100 jobs available per year, then by the end of 5 years, there are potentially as many as 2,000 anthropologists (assuming that none have left the job market) competing for 100 faculty positions. In addition, there are many individuals who re-enter the job market after landing their first tenure-track job to move to other types of intuitions or for a host of other reasons. Thus, there is simply no way that such a scenario is feasible long-term—yet this scenario is not far from reality. As shown in Fig 1, there has been a steady increase in the numbers of Anthropology PhDs over the past 30 years. Although a small fraction of these PhDs have found faculty employment in departments that have recently initiated doctoral programs or in extant programs that have expanded their faculty, new tenure-track positions in anthropology programs cannot keep pace with potential job seekers.

In addition to an overall decrease in new tenure-track positions [14], one must also consider that the numbers of doctorates awarded annually has been on upward trajectory for the past 30 years. When one also considers that the median age of college and university faculty now surpasses all other occupational groups [21] in the US, the net result is that longer faculty careers directly translate to a reduction in the rate of new hires. Consequently, academic job prospects for recent doctorates are dismal, not just in anthropology, but for most academic fields.

Not all doctoral programs have high or moderately high placement rates of graduates into faculty positions. It is evident from our study (Tables 1 and 2 and S1–S4 Tables) that certain programs command a high percentage of the total market share. We recognize that a limitation of our current study is that we cannot account for the number of graduate students admitted, nor graduate degrees conferred, by each department. Some smaller programs may in fact have excellent placement rates, but produce relatively few students, and therefore command a low percentage of the total market share. Conversely, programs that produce many graduates each year can command a high percentage of the market share with even moderate placement rates. Institutions that admit and produce large numbers of PhDs have a competitive advantage in terms of market share. We again emphasize that the “market share” concept does not equate to quality of education or research, nor does it necessarily determine or predict career outcomes. Nonetheless, it is imperative that prospective graduate students with aspirations of landing a tenure-track anthropology position are cognizant of departmental placement rates and consider this information when applying for graduate school.

Disparity in hiring females into tenure-track positions continues to be an issue. Males are consistently hired into academic anthropology positions at rates disproportionate to actual graduation rates. Quite simply, if >60% of all anthropology doctorates are female, then >60% of all new faculty hires also should be female. Our data suggest this is not the case and is an issue that needs to be addressed. It is beyond the scope of this paper to accurately address the root cause for this disparity. The reality—regardless of the root cause—is that between 2004 and 2014 males were being hired more often and into better-paying positions than their female counterparts [1, 28–29]. Our findings contribute to the larger discussion of gender inequity in anthropology, including two recent studies that have examined why females in anthropological archaeology apply for fewer grants than males after completing their PhDs [30] and the imbalance in the publication output of males versus females in anthropological archaeology [31]

## Conclusions

The outlook for tenure-track faculty positions in anthropology, as with many other disciplines, is somewhat bleak under our current models of graduate education. In sum, there are several structural components identified here that should make the would-be graduate student with faculty aspirations take pause. First, there is the nature of the job market, coupled with the production of too many PhDs competing for each position. Next, there is the fact that there are extreme disparities in the placement rate of certain programs over others in placing their graduates. Finally, despite some success in mediating gender disparities in hiring practices, these continue to persist throughout anthropology, with some subdisciplines being better than others. While in theory, anthropology is a forward-thinking and progressive discipline, which is certainly reflected in the content that we teach in our graduate seminars, in practice, the numbers point to a conservatism in what is valued in graduate education (i.e., academic success and a tenure-track position). In this way, anthropology is no different from any other discipline. We recognize that these revelations will be perhaps of no surprise to faculty in the trenches of departments everywhere. However, now they are quantified for all to see. Thus, we see this study as anthropology’s call to action, along with other similarly aligned disciplines, to do something about the situation we find our graduates and ourselves in now. The job market has changed dramatically in the last 20 years, and now is the time for the model we use to train future anthropologists to also change.

## Materials and methods

As described in detail previously [1] and quoted and paraphrased for context for the current study, the American Anthropological Association’s (AAA) *AnthroGuide* has been published annually since 1962, and it provides information on a broad range of anthropological institutions such as university departments, museums, and non-profit organizations. As each institution is responsible for providing their own information for inclusion in the *AnthroGuide*, the volume is not an exhaustive survey of all anthropological institutions [32]. However, most BA/BS, MA/MS, and PhD degree-granting US and Canadian institutions are represented in the guide.

For the purposes of this study, we analyzed data from degree-granting anthropology, and in rare cases archaeology, programs in the US. We did not include data for Canadian anthropology programs, museums, research institutes, nonprofits, private entities, or other organizations. For each degree-granting institution listed in the *AnthroGuide* [25], we recorded data for individuals listed as “faculty.” Faculty are considered to be tenure-track individuals who hold the title of “assistant,” “associate,” or “full” professor; however, some departments list individuals in permanent NTTF positions as faculty. In many cases, it appears that these are individuals who graduated from that department or are individuals who received positions via some sort of special hiring consideration (e.g., spousal hires). Information for people classified as anything other than “faculty” was not included in our database. These include categories such as lecturer, staff, post-doc, research staff, adjunct, affiliated, emeritus, museum professional, or anthropologists in other departments.

Relevant information for each individual recorded in our database included: first and last name, employment institution, graduate institution, year of graduation, subfield of expertise, apparent gender, highest degree offered by the employing institution, and current academic rank. The subfield of expertise was recorded based on research interests listed in the *AnthroGuide* [25] and faculty pages when interests in the guide were left blank or if clarification was needed.

We classified people into three subfield categories: archaeology, bioanthropology, and sociocultural anthropology. In our previous paper [1] we grouped archaeologists and bioarchaeologists together as a single unit (archaeologist). In the current study, bioarchaeologists are grouped with biological anthropologists which we believe better reflects this subfield. The sociocultural anthropology category includes linguists and anyone who is not an archaeologist or biological anthropologist.

Apparent gender was based on our perception of an individual’s first name as it was listed in the *AnthroGuide* [25], and verified, as necessary, through faculty webpages or other online resources when an individual’s name was gender-neutral or ambiguous. Academic rank categories we recorded included: assistant professor, associate professor, full professor, and “other” when a different title/rank was listed and the individual was listed as a regular faculty member.

Individuals with foreign (i.e., non-US) degrees represent a substantial portion of our academic population (ca. 5.5%). Although we recorded the institution, these individuals graduated from, for discussion purposes we classify all non-US derived doctoral degrees as “foreign”.

Some colleges and universities have separate anthropology and archaeology programs. We report information for these rare instances for the university as a whole, rather than listing them separately. Additionally, faculty employed within the City University of New York (CUNY) system are oftentimes cross-listed with several CUNY colleges (e.g., Hunter College, John Jay College, and/or Herbert H. Lehman College). For purposes of this study, the entire CUNY system is considered as a single entity.

As described in detail in our previous study [1], and quoted and paraphrased here for context for the current study, there are several limitations and inherent assumptions:

1. We can only see the end-result of the hiring process—who was hired, and where. We cannot account for differences in the quality and/or gender of applicants applying for academic positions in US anthropology departments.
2. The nature of our data limits us to a synchronic view of those who held a faculty appointment in a US anthropology department during the 2014–2015 academic year. We do not know how long a person has been employed at that particular department, how long they were on the job market, or how many previous academic positions the individual may (or may not) have had.
3. We do not include data for faculty at two-year colleges and technical schools. We recognize that many such schools employ both PhD and MA/MS-level anthropologists and archaeologists. However, our focus here is on tenure-track anthropology positions at BA/BS, MA/MS, and PhD granting institutions.
4. The number of PhDs produced each year by individual institutions is unknown. Institutions that produce larger numbers of PhDs will potentially have a competitive advantage in terms of market share.
5. The number of PhD students admitted each year that have tenure-track faculty job aspirations is unknown. Institutions that admit and produce large numbers of PhDs who desire this career path with have a competitive advantage in terms of market share.
6. We do not attempt to, nor can we account for, PhDs working outside of tenure-track anthropology programs in the US (e.g., industry, government, museum, NGOs, etc.) or anthropology PhDs who are faculty in non-anthropology departments or PhDs who have found academic employment in countries other than the US. Our focus here is solely on tenure-track faculty positions in US anthropology departments that offer BA/BS, MA/MS, and/or PhD degrees.
7. There is an inherent assumption that anthropology/archaeology faculty have PhDs in anthropology/archaeology. This is not always the case. Some archaeology faculty may have a PhD from a geoscience program, a biology-based program, a humanities-based archeology program, or even a general studies program. These individuals are rare, but it does explain why in a few cases we have listed a university as having produced a PhD when it is apparent that the institution does not have a PhD program in anthropology.
8. Not every academic program in the US and Canada reported data for the 2014–2015 *AnthroGuide* [25]. We attempted to identify PhD granting departments not listed and add them to our database. Faculty data that have been added to our database using departmental web pages include: Cornell University, Vanderbilt University, University of North Carolina at Chapel Hill, Stony Brook University, University of California at Berkeley, University of Illinois at Chicago, University of California Santa Cruz, and the Boston University Archaeology Department.

## Supporting information

S1 Table Summary of Anthropology department (all subfields) market share divided into 10-year increments beginning with 1974. (DOCX)

S2 Table Summary of archaeology market share divided into 10-year increments beginning with 1974. (DOCX)

S3 Table Summary of biological anthropology market share divided into 10-year increments beginning with 1974. (DOCX)

S4 Table Summary of sociocultural anthropology market share divided into 10-year increments beginning with 1974. (DOCX)

S5 Table Summary statistics for ranking and placement averages by subdiscipline for programs listed in S2–S4 Tables. (DOCX)

S6 Table US anthropology doctoral recipients by year relative to numbers of US anthropology doctorates who obtained anthropology faculty positions. (DOCX)
