# Supplementary material for: Market share and recent hiring trends in anthropology faculty positions
Source: PLoS One. 2018 Sep 12;13(9):e0202528. doi: 10.1371/journal.pone.0202528 (PMC6135356; doi:10.1371/journal.pone.0202528)
Supplement: S1 Table — (DOCX) [file pone.0202528.s001.docx]

**S1 Table. Summary of Anthropology department (all subfields) market share divided into 10-year increments (based on when the PhD was awarded, not when they obtained a faculty position) beginning with 1974**. Rankings are based on cumulative market share for the period 1994–2014.

|  | **All Years (3522)** | | **<1974 (161)** | | **1974-1983 (557)** | | **1984-1993 (734)** | | **1994-2003 (1045)** | | **2004-2014 (1025)** | | **20 Year Total (2070)** | |  |
| --- | --- | --- | --- | --- | --- | --- | --- | --- | --- | --- | --- | --- | --- | --- | --- |
| **University** | **n** | **%** | **n** | **%** | **n** | **%** | **n** | **%** | **n** | **%** | **n** | **%** | **n** | **%** | **Percentile** |
| Univ. Chicago | 220 | 6.2 | 24 | 14.8 | 37 | 6.6 | 44 | 6.0 | 54 | 5.2 | 61 | 6.0 | 115 | 5.6 | 95th |
| Foreign | 199 | 5.6 | 18 | 11.1 | 30 | 5.4 | 37 | 5.0 | 61 | 5.8 | 53 | 5.2 | 114 | 5.5 | 95th |
| Univ. Michigan, Ann Arbor | 187 | 5.3 | 7 | 4.3 | 32 | 5.7 | 42 | 5.7 | 57 | 5.5 | 49 | 4.8 | 106 | 5.1 | 95th |
| Univ. California, Berkeley | 172 | 4.9 | 12 | 7.4 | 27 | 4.8 | 43 | 5.9 | 50 | 4.8 | 40 | 3.9 | 90 | 4.3 | 95th |
| Harvard Univ. | 166 | 4.7 | 14 | 8.6 | 27 | 4.8 | 46 | 6.3 | 44 | 4.2 | 35 | 3.4 | 79 | 3.8 | 95th |
| Univ. Arizona | 105 | 3.0 | 4 | 2.5 | 15 | 2.7 | 23 | 3.1 | 28 | 2.7 | 35 | 3.4 | 63 | 3.0 | 95th |
| Univ. Texas, Austin | 76 | 2.2 | 1 | 0.6 | 11 | 2.0 | 12 | 1.6 | 16 | 1.5 | 36 | 3.5 | 52 | 2.5 | 90th |
| Univ. Pennsylvania | 97 | 2.8 | 2 | 1.2 | 22 | 3.9 | 22 | 3.0 | 23 | 2.2 | 28 | 2.7 | 51 | 2.5 | 90th |
| Univ. California, Los Angeles | 90 | 2.6 | 1 | 0.6 | 10 | 1.8 | 30 | 4.1 | 29 | 2.8 | 20 | 2.0 | 49 | 2.4 | 90th |
| New York Univ. | 62 | 1.8 | 1 | 0.6 | 7 | 1.3 | 6 | 0.8 | 16 | 1.5 | 32 | 3.1 | 48 | 2.3 | 90th |
| Yale Univ. | 73 | 2.1 | 1 | 0.6 | 15 | 2.7 | 13 | 1.8 | 26 | 2.5 | 18 | 1.8 | 44 | 2.1 | 90th |
| Stanford Univ. | 76 | 2.2 | 2 | 1.2 | 15 | 2.7 | 18 | 2.5 | 28 | 2.7 | 13 | 1.3 | 41 | 2.0 | 75th |
| Columbia Univ. | 89 | 2.5 | 11 | 6.8 | 22 | 3.9 | 17 | 2.3 | 17 | 1.6 | 22 | 2.1 | 39 | 1.9 | 75th |
| Univ. New Mexico | 55 | 1.6 | 0 | 0.0 | 2 | 0.4 | 14 | 1.9 | 19 | 1.8 | 20 | 2.0 | 39 | 1.9 | 75th |
| Arizona St. Univ. | 50 | 1.4 | 1 | 0.6 | 4 | 0.7 | 6 | 0.8 | 25 | 2.4 | 14 | 1.4 | 39 | 1.9 | 75th |
| City Univ. New York | 63 | 1.8 | 1 | 0.6 | 6 | 1.1 | 18 | 2.5 | 20 | 1.9 | 18 | 1.8 | 38 | 1.8 | 75th |
| Indiana Univ., Bloomington | 68 | 1.9 | 2 | 1.2 | 14 | 2.5 | 15 | 2.0 | 20 | 1.9 | 17 | 1.7 | 37 | 1.8 | 75th |
| Univ. Florida | 56 | 1.6 | 0 | 0.0 | 9 | 1.6 | 11 | 1.5 | 20 | 1.9 | 16 | 1.6 | 36 | 1.7 | 75th |
| Univ. Illinois, Urbana-Champaign | 64 | 1.8 | 5 | 3.1 | 12 | 2.2 | 13 | 1.8 | 22 | 2.1 | 12 | 1.2 | 34 | 1.6 | 75th |
| Univ. Washington | 52 | 1.5 | 2 | 1.2 | 8 | 1.4 | 9 | 1.2 | 17 | 1.6 | 16 | 1.6 | 33 | 1.6 | 75th |
| Emory Univ. | 33 | 0.9 | 0 | 0.0 | 0 | 0.0 | 1 | 0.1 | 14 | 1.3 | 18 | 1.8 | 32 | 1.5 | 75th |
| Univ. Wisconsin, Madison | 51 | 1.4 | 5 | 3.1 | 4 | 0.7 | 13 | 1.8 | 17 | 1.6 | 12 | 1.2 | 29 | 1.4 | 75th |
| Univ. California, Santa Barbara | 57 | 1.6 | 1 | 0.6 | 11 | 2.0 | 17 | 2.3 | 14 | 1.3 | 14 | 1.4 | 28 | 1.4 | 75th |
| Cornell Univ. | 49 | 1.4 | 3 | 1.9 | 7 | 1.3 | 11 | 1.5 | 14 | 1.3 | 14 | 1.4 | 28 | 1.4 | 75th |
| Univ. California, Davis | 39 | 1.1 | 0 | 0.0 | 6 | 1.1 | 6 | 0.8 | 15 | 1.4 | 12 | 1.2 | 27 | 1.3 | 75th |
| Univ. North Carolina, Chapel Hill | 37 | 1.0 | 2 | 1.2 | 4 | 0.7 | 4 | 0.5 | 14 | 1.3 | 13 | 1.3 | 27 | 1.3 | 75th |
| Washington Univ., St. Louis | 35 | 1.0 | 0 | 0.0 | 4 | 0.7 | 6 | 0.8 | 15 | 1.4 | 10 | 1.0 | 25 | 1.2 | 75th |
| Univ. Pittsburgh | 37 | 1.0 | 2 | 1.2 | 5 | 0.9 | 6 | 0.8 | 13 | 1.2 | 11 | 1.1 | 24 | 1.2 | 75th |
| Univ. Georgia | 29 | 0.8 | 0 | 0.0 | 4 | 0.7 | 1 | 0.1 | 13 | 1.2 | 11 | 1.1 | 24 | 1.2 | 75th |
| Univ. Virginia | 29 | 0.8 | 0 | 0.0 | 2 | 0.4 | 3 | 0.4 | 14 | 1.3 | 10 | 1.0 | 24 | 1.2 | 75th |
| Pennsylvania St. Univ. | 47 | 1.3 | 2 | 1.2 | 12 | 2.2 | 10 | 1.4 | 12 | 1.1 | 11 | 1.1 | 23 | 1.1 | 50th |
| SUNY Stony Brook | 33 | 0.9 | 0 | 0.0 | 3 | 0.5 | 8 | 1.1 | 13 | 1.2 | 9 | 0.9 | 22 | 1.1 | 50th |
| Univ. California, Santa Cruz | 24 | 0.7 | 0 | 0.0 | 1 | 0.2 | 1 | 0.1 | 7 | 0.7 | 15 | 1.5 | 22 | 1.1 | 50th |
| Duke Univ. | 31 | 0.9 | 1 | 0.6 | 4 | 0.7 | 5 | 0.7 | 9 | 0.9 | 12 | 1.2 | 21 | 1.0 | 50th |
| Northwestern Univ. | 53 | 1.5 | 4 | 2.5 | 12 | 2.2 | 17 | 2.3 | 5 | 0.5 | 15 | 1.5 | 20 | 1.0 | 50th |
| Univ. Tennessee, Knoxville | 28 | 0.8 | 0 | 0.0 | 3 | 0.5 | 5 | 0.7 | 13 | 1.2 | 7 | 0.7 | 20 | 1.0 | 50th |
| Univ. California, San Diego | 35 | 1.0 | 0 | 0.0 | 5 | 0.9 | 11 | 1.5 | 12 | 1.1 | 7 | 0.7 | 19 | 0.9 | 50th |
| Johns Hopkins Univ. | 29 | 0.8 | 0 | 0.0 | 4 | 0.7 | 6 | 0.8 | 12 | 1.1 | 7 | 0.7 | 19 | 0.9 | 50th |
| Rutgers Univ. | 24 | 0.7 | 0 | 0.0 | 1 | 0.2 | 4 | 0.5 | 7 | 0.7 | 12 | 1.2 | 19 | 0.9 | 50th |
| Tulane Univ. | 24 | 0.7 | 1 | 0.6 | 1 | 0.2 | 3 | 0.4 | 8 | 0.8 | 11 | 1.1 | 19 | 0.9 | 50th |
| Univ. Massachusetts, Amherst | 47 | 1.3 | 2 | 1.2 | 13 | 2.3 | 14 | 1.9 | 12 | 1.1 | 6 | 0.6 | 18 | 0.9 | 50th |
| Michigan St. Univ. | 31 | 0.9 | 2 | 1.2 | 3 | 0.5 | 8 | 1.1 | 7 | 0.7 | 11 | 1.1 | 18 | 0.9 | 50th |
| Texas A&M Univ. | 18 | 0.5 | 0 | 0.0 | 0 | 0.0 | 0 | 0.0 | 7 | 0.7 | 11 | 1.1 | 18 | 0.9 | 50th |
| Univ. Colorado, Boulder | 33 | 0.9 | 2 | 1.2 | 9 | 1.6 | 5 | 0.7 | 8 | 0.8 | 9 | 0.9 | 17 | 0.8 | 50th |
| Ohio St. Univ. | 25 | 0.7 | 0 | 0.0 | 3 | 0.5 | 5 | 0.7 | 8 | 0.8 | 9 | 0.9 | 17 | 0.8 | 50th |
| Univ. Oregon | 26 | 0.7 | 4 | 2.5 | 2 | 0.4 | 4 | 0.5 | 8 | 0.8 | 8 | 0.8 | 16 | 0.8 | 50th |
| Washington St. Univ. | 28 | 0.8 | 0 | 0.0 | 7 | 1.3 | 6 | 0.8 | 4 | 0.4 | 11 | 1.1 | 15 | 0.7 | 50th |
| SUNY Albany | 23 | 0.7 | 0 | 0.0 | 3 | 0.5 | 5 | 0.7 | 7 | 0.7 | 8 | 0.8 | 15 | 0.7 | 50th |
| Temple Univ. | 22 | 0.6 | 0 | 0.0 | 1 | 0.2 | 6 | 0.8 | 5 | 0.5 | 10 | 1.0 | 15 | 0.7 | 50th |
| Brown Univ. | 28 | 0.8 | 0 | 0.0 | 6 | 1.1 | 8 | 1.1 | 6 | 0.6 | 8 | 0.8 | 14 | 0.7 | 50th |
| SUNY Binghamton | 33 | 0.9 | 0 | 0.0 | 7 | 1.3 | 13 | 1.8 | 6 | 0.6 | 7 | 0.7 | 13 | 0.6 | 50th |
| Univ. Kentucky | 17 | 0.5 | 1 | 0.6 | 0 | 0.0 | 4 | 0.5 | 1 | 0.1 | 11 | 1.1 | 12 | 0.6 | 50th |
| Princeton Univ. | 21 | 0.6 | 0 | 0.0 | 7 | 1.3 | 3 | 0.4 | 7 | 0.7 | 4 | 0.4 | 11 | 0.5 | 50th |
| Southern Methodist Univ. | 21 | 0.6 | 3 | 1.9 | 4 | 0.7 | 3 | 0.4 | 7 | 0.7 | 4 | 0.4 | 11 | 0.5 | 50th |
| SUNY Buffalo | 21 | 0.6 | 0 | 0.0 | 5 | 0.9 | 5 | 0.7 | 6 | 0.6 | 5 | 0.5 | 11 | 0.5 | 50th |
| Syracuse Univ. | 16 | 0.5 | 1 | 0.6 | 2 | 0.4 | 2 | 0.3 | 5 | 0.5 | 6 | 0.6 | 11 | 0.5 | 50th |
| Univ. Minnesota | 16 | 0.5 | 1 | 0.6 | 3 | 0.5 | 1 | 0.1 | 5 | 0.5 | 6 | 0.6 | 11 | 0.5 | 50th |
| Boston Univ. | 18 | 0.5 | 0 | 0.0 | 5 | 0.9 | 3 | 0.4 | 4 | 0.4 | 6 | 0.6 | 10 | 0.5 | 25th |
| Southern Illinois Univ., Carbondale | 15 | 0.4 | 0 | 0.0 | 3 | 0.5 | 2 | 0.3 | 4 | 0.4 | 6 | 0.6 | 10 | 0.5 | 25th |
| Univ. California, Irvine | 13 | 0.4 | 0 | 0.0 | 2 | 0.4 | 1 | 0.1 | 4 | 0.4 | 6 | 0.6 | 10 | 0.5 | 25th |
| Univ. Hawaii | 12 | 0.3 | 0 | 0.0 | 0 | 0.0 | 2 | 0.3 | 5 | 0.5 | 5 | 0.5 | 10 | 0.5 | 25th |
| Univ. Missouri | 17 | 0.5 | 1 | 0.6 | 3 | 0.5 | 4 | 0.5 | 9 | 0.9 | 0 | 0.0 | 9 | 0.4 | 25th |
| Univ. California, Riverside | 14 | 0.4 | 0 | 0.0 | 4 | 0.7 | 1 | 0.1 | 3 | 0.3 | 6 | 0.6 | 9 | 0.4 | 25th |
| American Univ. | 18 | 0.5 | 1 | 0.6 | 5 | 0.9 | 4 | 0.5 | 4 | 0.4 | 4 | 0.4 | 8 | 0.4 | 25th |
| Univ. Oklahoma | 15 | 0.4 | 0 | 0.0 | 2 | 0.4 | 5 | 0.7 | 7 | 0.7 | 1 | 0.1 | 8 | 0.4 | 25th |
| Vanderbilt Univ. | 9 | 0.3 | 0 | 0.0 | 0 | 0.0 | 1 | 0.1 | 6 | 0.6 | 2 | 0.2 | 8 | 0.4 | 25th |
| Univ. Illinois, Chicago | 8 | 0.2 | 0 | 0.0 | 0 | 0.0 | 0 | 0.0 | 0 | 0.0 | 8 | 0.8 | 8 | 0.4 | 25th |
| Univ. Iowa | 10 | 0.3 | 1 | 0.6 | 1 | 0.2 | 1 | 0.1 | 5 | 0.5 | 2 | 0.2 | 7 | 0.3 | 25th |
| Univ. Southern California | 8 | 0.2 | 0 | 0.0 | 1 | 0.2 | 0 | 0.0 | 5 | 0.5 | 2 | 0.2 | 7 | 0.3 | 25th |
| New School | 15 | 0.4 | 0 | 0.0 | 3 | 0.5 | 6 | 0.8 | 2 | 0.2 | 4 | 0.4 | 6 | 0.3 | 25th |
| Rice Univ. | 9 | 0.3 | 0 | 0.0 | 1 | 0.2 | 2 | 0.3 | 1 | 0.1 | 5 | 0.5 | 6 | 0.3 | 25th |
| Case Western Reserve Univ. | 9 | 0.3 | 0 | 0.0 | 2 | 0.4 | 2 | 0.3 | 5 | 0.5 | 0 | 0.0 | 5 | 0.2 | 25th |
| Florida St. Univ. | 6 | 0.2 | 1 | 0.6 | 0 | 0.0 | 0 | 0.0 | 1 | 0.1 | 4 | 0.4 | 5 | 0.2 | 25th |
| Univ. Alabama | 5 | 0.1 | 0 | 0.0 | 0 | 0.0 | 0 | 0.0 | 0 | 0.0 | 5 | 0.5 | 5 | 0.2 | 25th |
| Univ. Arkansas | 5 | 0.1 | 0 | 0.0 | 0 | 0.0 | 0 | 0.0 | 1 | 0.1 | 4 | 0.4 | 5 | 0.2 | 25th |
| Univ. Utah | 15 | 0.4 | 1 | 0.6 | 3 | 0.5 | 7 | 1.0 | 3 | 0.3 | 1 | 0.1 | 4 | 0.2 | 25th |
| Univ. Connecticut | 11 | 0.3 | 0 | 0.0 | 6 | 1.1 | 1 | 0.1 | 2 | 0.2 | 2 | 0.2 | 4 | 0.2 | 25th |
| Univ. Kansas | 11 | 0.3 | 1 | 0.6 | 2 | 0.4 | 4 | 0.5 | 3 | 0.3 | 1 | 0.1 | 4 | 0.2 | 25th |
| Kent St. Univ. | 6 | 0.2 | 0 | 0.0 | 0 | 0.0 | 2 | 0.3 | 2 | 0.2 | 2 | 0.2 | 4 | 0.2 | 25th |
| Univ. Nebraska, Lincoln | 4 | 0.1 | 0 | 0.0 | 0 | 0.0 | 0 | 0.0 | 0 | 0.0 | 4 | 0.4 | 4 | 0.2 | 25th |
| Univ. Nevada, Reno | 4 | 0.1 | 0 | 0.0 | 0 | 0.0 | 0 | 0.0 | 3 | 0.3 | 1 | 0.1 | 4 | 0.2 | 25th |
| Univ. South Florida | 4 | 0.1 | 0 | 0.0 | 0 | 0.0 | 0 | 0.0 | 1 | 0.1 | 3 | 0.3 | 4 | 0.2 | 25th |
| Brandeis Univ. | 8 | 0.2 | 0 | 0.0 | 3 | 0.5 | 2 | 0.3 | 2 | 0.2 | 1 | 0.1 | 3 | 0.1 | 10th |
| Purdue Univ. | 5 | 0.1 | 0 | 0.0 | 0 | 0.0 | 2 | 0.3 | 2 | 0.2 | 1 | 0.1 | 3 | 0.1 | 10th |
| Univ. Maryland | 6 | 0.2 | 3 | 1.9 | 0 | 0.0 | 0 | 0.0 | 0 | 0.0 | 3 | 0.3 | 3 | 0.1 | 10th |
| Univ. Nevada, Las Vegas | 3 | 0.1 | 0 | 0.0 | 0 | 0.0 | 0 | 0.0 | 0 | 0.0 | 3 | 0.3 | 3 | 0.1 | 10th |
| Univ. Rochester | 12 | 0.3 | 4 | 2.5 | 4 | 0.7 | 1 | 0.1 | 3 | 0.3 | 0 | 0.0 | 3 | 0.1 | 10th |
| Univ. Wisconsin, Milwaukee | 8 | 0.2 | 0 | 0.0 | 4 | 0.7 | 1 | 0.1 | 2 | 0.2 | 1 | 0.1 | 3 | 0.1 | 10th |
| Catholic Univ. America | 7 | 0.2 | 1 | 0.6 | 3 | 0.5 | 1 | 0.1 | 2 | 0.2 | 0 | 0.0 | 2 | 0.1 | 10th |
| Univ. Alaska, Fairbanks | 3 | 0.1 | 0 | 0.0 | 0 | 0.0 | 1 | 0.1 | 1 | 0.1 | 1 | 0.1 | 2 | 0.1 | 10th |
| Univ. South Carolina | 2 | 0.1 | 0 | 0.0 | 0 | 0.0 | 0 | 0.0 | 2 | 0.2 | 0 | 0.0 | 2 | 0.1 | 10th |
| Univ. Wyoming | 2 | 0.1 | 0 | 0.0 | 0 | 0.0 | 0 | 0.0 | 0 | 0.0 | 2 | 0.2 | 2 | 0.1 | 10th |
| Boston Coll. | 1 | 0.0 | 0 | 0.0 | 0 | 0.0 | 0 | 0.0 | 0 | 0.0 | 1 | 0.1 | 1 | 0.0 | 10th |
| Bryn Mawr Coll. | 8 | 0.2 | 0 | 0.0 | 5 | 0.9 | 2 | 0.3 | 1 | 0.1 | 0 | 0.0 | 1 | 0.0 | 10th |
| Clark Univ. | 2 | 0.1 | 0 | 0.0 | 0 | 0.0 | 1 | 0.1 | 1 | 0.1 | 0 | 0.0 | 1 | 0.0 | 10th |
| Coll. William and Mary | 1 | 0.0 | 0 | 0.0 | 0 | 0.0 | 0 | 0.0 | 0 | 0.0 | 1 | 0.1 | 1 | 0.0 | 10th |
| Colorado St. Univ. | 1 | 0.0 | 0 | 0.0 | 0 | 0.0 | 0 | 0.0 | 0 | 0.0 | 1 | 0.1 | 1 | 0.0 | 10th |
| Louisiana St. Univ. | 1 | 0.0 | 0 | 0.0 | 0 | 0.0 | 0 | 0.0 | 1 | 0.1 | 0 | 0.0 | 1 | 0.0 | 10th |
| Massachusetts Institute Technology | 3 | 0.1 | 0 | 0.0 | 0 | 0.0 | 2 | 0.3 | 1 | 0.1 | 0 | 0.0 | 1 | 0.0 | 10th |
| Montana St. Univ. | 1 | 0.0 | 0 | 0.0 | 0 | 0.0 | 0 | 0.0 | 1 | 0.1 | 0 | 0.0 | 1 | 0.0 | 10th |
| North Carolina St. Univ. | 2 | 0.1 | 0 | 0.0 | 0 | 0.0 | 1 | 0.1 | 0 | 0.0 | 1 | 0.1 | 1 | 0.0 | 10th |
| Rensselaer Polytechnic Institute | 2 | 0.1 | 1 | 0.6 | 0 | 0.0 | 0 | 0.0 | 0 | 0.0 | 1 | 0.1 | 1 | 0.0 | 10th |
| Texas St. Univ., San Marcos | 1 | 0.0 | 0 | 0.0 | 0 | 0.0 | 0 | 0.0 | 0 | 0.0 | 1 | 0.1 | 1 | 0.0 | 10th |
| Union Institute and Univ. | 1 | 0.0 | 0 | 0.0 | 0 | 0.0 | 0 | 0.0 | 1 | 0.1 | 0 | 0.0 | 1 | 0.0 | 10th |
| Univ. California, San Francisco | 2 | 0.1 | 0 | 0.0 | 0 | 0.0 | 1 | 0.1 | 0 | 0.0 | 1 | 0.1 | 1 | 0.0 | 10th |
| Univ. Cincinnati | 1 | 0.0 | 0 | 0.0 | 0 | 0.0 | 0 | 0.0 | 1 | 0.1 | 0 | 0.0 | 1 | 0.0 | 10th |
| Univ. Delaware | 2 | 0.1 | 0 | 0.0 | 1 | 0.2 | 0 | 0.0 | 1 | 0.1 | 0 | 0.0 | 1 | 0.0 | 10th |
| Univ. Texas, San Antonio | 1 | 0.0 | 0 | 0.0 | 0 | 0.0 | 0 | 0.0 | 0 | 0.0 | 1 | 0.1 | 1 | 0.0 | 10th |
| Wayne St. Univ. | 4 | 0.1 | 0 | 0.0 | 1 | 0.2 | 2 | 0.3 | 0 | 0.0 | 1 | 0.1 | 1 | 0.0 | 10th |
| Wesleyan | 1 | 0.0 | 0 | 0.0 | 0 | 0.0 | 0 | 0.0 | 1 | 0.1 | 0 | 0.0 | 1 | 0.0 | 10th |
