# Supplementary material for: Market share and recent hiring trends in anthropology faculty positions
Source: PLoS One. 2018 Sep 12;13(9):e0202528. doi: 10.1371/journal.pone.0202528 (PMC6135356; doi:10.1371/journal.pone.0202528)
Supplement: S2 Table — (DOCX) [file pone.0202528.s002.docx]

**S2 Table. Summary of Archaeology market share divided into 10-year increments (based on when the PhD was awarded, not when they obtained a faculty position) beginning with 1974.** Rankings are based on cumulative market share for the period 1994–2014.

| **University** | **All Years (897)** | | **<1974 (33)** | | **1974-1983 (159)** | | **1984-1993 (225)** | | **1994-2003 (247)** | | **2004-2014 (233)** | | **20 Year Total (480)** | |  |
| --- | --- | --- | --- | --- | --- | --- | --- | --- | --- | --- | --- | --- | --- | --- | --- |
|  | **n** | **%** | **n** | **%** | **n** | **%** | **n** | **%** | **n** | **%** | **n** | **%** | **n** | **%** | **Percentile** |
| Univ. Michigan, Ann Arbor | 64 | 7.0 | 4 | 10.8 | 10 | 6.0 | 19 | 8.2 | 16 | 6.5 | 15 | 6.4 | 31 | 6.5 | 95th |
| Foreign | 56 | 6.1 | 1 | 2.7 | 11 | 6.5 | 14 | 6.1 | 14 | 5.7 | 16 | 6.9 | 30 | 6.3 | 95th |
| Univ. California, Berkeley | 41 | 4.5 | 2 | 5.4 | 5 | 3.0 | 9 | 3.9 | 12 | 4.9 | 13 | 5.6 | 25 | 5.2 | 95th |
| Univ. Arizona | 51 | 5.6 | 3 | 8.1 | 10 | 6.0 | 14 | 6.1 | 12 | 4.9 | 12 | 5.2 | 24 | 5.0 | 95th |
| Harvard Univ. | 43 | 4.7 | 4 | 10.8 | 11 | 6.5 | 10 | 4.3 | 11 | 4.5 | 7 | 3.0 | 18 | 3.8 | 90th |
| Arizona St. Univ. | 25 | 2.7 | 0 | 0.0 | 4 | 2.4 | 4 | 1.7 | 12 | 4.9 | 5 | 2.1 | 17 | 3.5 | 90th |
| Univ. Pennsylvania | 40 | 4.4 | 1 | 2.7 | 11 | 6.5 | 12 | 5.2 | 8 | 3.2 | 8 | 3.4 | 16 | 3.3 | 90th |
| Texas A&M Univ. | 15 | 1.6 | 0 | 0.0 | 0 | 0.0 | 0 | 0.0 | 6 | 2.4 | 9 | 3.9 | 15 | 3.1 | 90th |
| Univ. California, Los Angeles | 25 | 2.7 | 0 | 0.0 | 4 | 2.4 | 9 | 3.9 | 7 | 2.8 | 5 | 2.1 | 12 | 2.5 | 75th |
| Univ. Chicago | 25 | 2.7 | 3 | 8.1 | 3 | 1.8 | 7 | 3.0 | 4 | 1.6 | 8 | 3.4 | 12 | 2.5 | 75th |
| Univ. Wisconsin, Madison | 18 | 2.0 | 2 | 5.4 | 1 | 0.6 | 3 | 1.3 | 9 | 3.6 | 3 | 1.3 | 12 | 2.5 | 75th |
| Univ. Florida | 21 | 2.3 | 0 | 0.0 | 5 | 3.0 | 5 | 2.2 | 8 | 3.2 | 3 | 1.3 | 11 | 2.3 | 75th |
| Univ. California, Santa Barbara | 26 | 2.8 | 0 | 0.0 | 5 | 3.0 | 11 | 4.8 | 6 | 2.4 | 4 | 1.7 | 10 | 2.1 | 75th |
| Univ. New Mexico | 21 | 2.3 | 0 | 0.0 | 0 | 0.0 | 11 | 4.8 | 3 | 1.2 | 7 | 3.0 | 10 | 2.1 | 75th |
| Univ. Texas, Austin | 16 | 1.7 | 1 | 2.7 | 4 | 2.4 | 1 | 0.4 | 4 | 1.6 | 6 | 2.6 | 10 | 2.1 | 75th |
| Univ. Pittsburgh | 13 | 1.4 | 0 | 0.0 | 0 | 0.0 | 3 | 1.3 | 6 | 2.4 | 4 | 1.7 | 10 | 2.1 | 75th |
| Univ. Virginia | 10 | 1.1 | 0 | 0.0 | 0 | 0.0 | 0 | 0.0 | 6 | 2.4 | 4 | 1.7 | 10 | 2.1 | 75th |
| Southern Methodist Univ. | 16 | 1.7 | 1 | 2.7 | 4 | 2.4 | 2 | 0.9 | 5 | 2.0 | 4 | 1.7 | 9 | 1.9 | 75th |
| Univ. Massachusetts, Amherst | 15 | 1.6 | 0 | 0.0 | 5 | 3.0 | 2 | 0.9 | 6 | 2.4 | 2 | 0.9 | 8 | 1.7 | 75th |
| Univ. Washington | 13 | 1.4 | 0 | 0.0 | 1 | 0.6 | 4 | 1.7 | 4 | 1.6 | 4 | 1.7 | 8 | 1.7 | 75th |
| Univ. North Carolina, Chapel Hill | 12 | 1.3 | 0 | 0.0 | 1 | 0.6 | 3 | 1.3 | 3 | 1.2 | 5 | 2.1 | 8 | 1.7 | 75th |
| Northwestern Univ. | 18 | 2.0 | 0 | 0.0 | 4 | 2.4 | 7 | 3.0 | 1 | 0.4 | 6 | 2.6 | 7 | 1.5 | 50th |
| Pennsylvania St. Univ. | 15 | 1.6 | 0 | 0.0 | 4 | 2.4 | 4 | 1.7 | 5 | 2.0 | 2 | 0.9 | 7 | 1.5 | 50th |
| Michigan St. Univ. | 12 | 1.3 | 1 | 2.7 | 1 | 0.6 | 3 | 1.3 | 2 | 0.8 | 5 | 2.1 | 7 | 1.5 | 50th |
| Washington Univ., St. Louis | 12 | 1.3 | 0 | 0.0 | 2 | 1.2 | 3 | 1.3 | 5 | 2.0 | 2 | 0.9 | 7 | 1.5 | 50th |
| Univ. Georgia | 11 | 1.2 | 0 | 0.0 | 4 | 2.4 | 0 | 0.0 | 5 | 2.0 | 2 | 0.9 | 7 | 1.5 | 50th |
| Washington St. Univ. | 11 | 1.2 | 0 | 0.0 | 2 | 1.2 | 2 | 0.9 | 2 | 0.8 | 5 | 2.1 | 7 | 1.5 | 50th |
| Vanderbilt Univ. | 8 | 0.9 | 0 | 0.0 | 0 | 0.0 | 1 | 0.4 | 6 | 2.4 | 1 | 0.4 | 7 | 1.5 | 50th |
| Univ. Illinois, Urbana-Champaign | 16 | 1.7 | 2 | 5.4 | 3 | 1.8 | 5 | 2.2 | 5 | 2.0 | 1 | 0.4 | 6 | 1.3 | 50th |
| Syracuse Univ. | 7 | 0.8 | 0 | 0.0 | 0 | 0.0 | 1 | 0.4 | 1 | 0.4 | 5 | 2.1 | 6 | 1.3 | 50th |
| Stanford Univ. | 6 | 0.7 | 0 | 0.0 | 0 | 0.0 | 0 | 0.0 | 3 | 1.2 | 3 | 1.3 | 6 | 1.3 | 50th |
| SUNY Binghamton | 18 | 2.0 | 0 | 0.0 | 4 | 2.4 | 9 | 3.9 | 4 | 1.6 | 1 | 0.4 | 5 | 1.0 | 50th |
| Yale Univ. | 16 | 1.7 | 0 | 0.0 | 4 | 2.4 | 7 | 3.0 | 2 | 0.8 | 3 | 1.3 | 5 | 1.0 | 50th |
| Brown Univ. | 12 | 1.3 | 0 | 0.0 | 3 | 1.8 | 4 | 1.7 | 3 | 1.2 | 2 | 0.9 | 5 | 1.0 | 50th |
| Tulane Univ. | 10 | 1.1 | 1 | 2.7 | 1 | 0.6 | 3 | 1.3 | 3 | 1.2 | 2 | 0.9 | 5 | 1.0 | 50th |
| Univ. Colorado, Boulder | 9 | 1.0 | 0 | 0.0 | 4 | 2.4 | 0 | 0.0 | 2 | 0.8 | 3 | 1.3 | 5 | 1.0 | 50th |
| Boston Univ. | 7 | 0.8 | 0 | 0.0 | 1 | 0.6 | 1 | 0.4 | 1 | 0.4 | 4 | 1.7 | 5 | 1.0 | 50th |
| Univ. California, Riverside | 7 | 0.8 | 0 | 0.0 | 2 | 1.2 | 0 | 0.0 | 1 | 0.4 | 4 | 1.7 | 5 | 1.0 | 50th |
| Southern Illinois Univ., Carbondale | 8 | 0.9 | 0 | 0.0 | 3 | 1.8 | 1 | 0.4 | 1 | 0.4 | 3 | 1.3 | 4 | 0.8 | 25th |
| Univ. Tennessee, Knoxville | 7 | 0.8 | 0 | 0.0 | 0 | 0.0 | 3 | 1.3 | 4 | 1.6 | 0 | 0.0 | 4 | 0.8 | 25th |
| Univ. Kentucky | 5 | 0.5 | 0 | 0.0 | 0 | 0.0 | 1 | 0.4 | 0 | 0.0 | 4 | 1.7 | 4 | 0.8 | 25th |
| Univ. Oregon | 5 | 0.5 | 1 | 2.7 | 0 | 0.0 | 0 | 0.0 | 3 | 1.2 | 1 | 0.4 | 4 | 0.8 | 25th |
| Indiana Univ., Bloomington | 10 | 1.1 | 1 | 2.7 | 3 | 1.8 | 3 | 1.3 | 2 | 0.8 | 1 | 0.4 | 3 | 0.6 | 25th |
| Columbia Univ. | 8 | 0.9 | 2 | 5.4 | 3 | 1.8 | 0 | 0.0 | 2 | 0.8 | 1 | 0.4 | 3 | 0.6 | 25th |
| SUNY Buffalo | 6 | 0.7 | 0 | 0.0 | 1 | 0.6 | 2 | 0.9 | 1 | 0.4 | 2 | 0.9 | 3 | 0.6 | 25th |
| City Univ. New York | 4 | 0.4 | 0 | 0.0 | 0 | 0.0 | 1 | 0.4 | 2 | 0.8 | 1 | 0.4 | 3 | 0.6 | 25th |
| Univ. Hawaii | 3 | 0.3 | 0 | 0.0 | 0 | 0.0 | 0 | 0.0 | 1 | 0.4 | 2 | 0.9 | 3 | 0.6 | 25th |
| Univ. Illinois, Chicago | 3 | 0.3 | 0 | 0.0 | 0 | 0.0 | 0 | 0.0 | 0 | 0.0 | 3 | 1.3 | 3 | 0.6 | 25th |
| Univ. Nevada, Reno | 3 | 0.3 | 0 | 0.0 | 0 | 0.0 | 0 | 0.0 | 2 | 0.8 | 1 | 0.4 | 3 | 0.6 | 25th |
| Cornell Univ. | 4 | 0.4 | 0 | 0.0 | 1 | 0.6 | 1 | 0.4 | 2 | 0.8 | 0 | 0.0 | 2 | 0.4 | 10th |
| Univ. California, Davis | 4 | 0.4 | 0 | 0.0 | 1 | 0.6 | 1 | 0.4 | 0 | 0.0 | 2 | 0.9 | 2 | 0.4 | 10th |
| Univ. California, Santa Cruz | 2 | 0.2 | 0 | 0.0 | 0 | 0.0 | 0 | 0.0 | 0 | 0.0 | 2 | 0.9 | 2 | 0.4 | 10th |
| Univ. Iowa | 2 | 0.2 | 0 | 0.0 | 0 | 0.0 | 0 | 0.0 | 1 | 0.4 | 1 | 0.4 | 2 | 0.4 | 10th |
| Univ. Minnesota | 4 | 0.4 | 0 | 0.0 | 1 | 0.6 | 1 | 0.4 | 1 | 0.4 | 1 | 0.4 | 2 | 0.4 | 10th |
| Univ. Missouri | 9 | 1.0 | 1 | 2.7 | 3 | 1.8 | 3 | 1.3 | 2 | 0.8 | 0 | 0.0 | 2 | 0.4 | 10th |
| Univ. Utah | 9 | 1.0 | 1 | 2.7 | 0 | 0.0 | 6 | 2.6 | 1 | 0.4 | 1 | 0.4 | 2 | 0.4 | 10th |
| Univ. Wisconsin, Milwaukee | 5 | 0.5 | 0 | 0.0 | 2 | 1.2 | 1 | 0.4 | 2 | 0.8 | 0 | 0.0 | 2 | 0.4 | 10th |
| Univ. Wyoming | 2 | 0.2 | 0 | 0.0 | 0 | 0.0 | 0 | 0.0 | 0 | 0.0 | 2 | 0.9 | 2 | 0.4 | 10th |
| Bryn Mawr Coll. | 3 | 0.3 | 0 | 0.0 | 1 | 0.6 | 1 | 0.4 | 1 | 0.4 | 0 | 0.0 | 1 | 0.2 | 10th |
| Catholic Univ. America | 4 | 0.4 | 0 | 0.0 | 3 | 1.8 | 0 | 0.0 | 1 | 0.4 | 0 | 0.0 | 1 | 0.2 | 10th |
| Coll. William and Mary | 1 | 0.1 | 0 | 0.0 | 0 | 0.0 | 0 | 0.0 | 0 | 0.0 | 1 | 0.4 | 1 | 0.2 | 10th |
| Florida St. Univ. | 2 | 0.2 | 1 | 2.7 | 0 | 0.0 | 0 | 0.0 | 0 | 0.0 | 1 | 0.4 | 1 | 0.2 | 10th |
| New York Univ. | 4 | 0.4 | 0 | 0.0 | 1 | 0.6 | 2 | 0.9 | 1 | 0.4 | 0 | 0.0 | 1 | 0.2 | 10th |
| Rutgers Univ. | 2 | 0.2 | 0 | 0.0 | 0 | 0.0 | 1 | 0.4 | 1 | 0.4 | 0 | 0.0 | 1 | 0.2 | 10th |
| SUNY Albany | 3 | 0.3 | 0 | 0.0 | 1 | 0.6 | 1 | 0.4 | 1 | 0.4 | 0 | 0.0 | 1 | 0.2 | 10th |
| SUNY Stony Brook | 1 | 0.1 | 0 | 0.0 | 0 | 0.0 | 0 | 0.0 | 0 | 0.0 | 1 | 0.4 | 1 | 0.2 | 10th |
| Temple Univ. | 2 | 0.2 | 0 | 0.0 | 0 | 0.0 | 1 | 0.4 | 0 | 0.0 | 1 | 0.4 | 1 | 0.2 | 10th |
| Union Institute and Univ. | 1 | 0.1 | 0 | 0.0 | 0 | 0.0 | 0 | 0.0 | 1 | 0.4 | 0 | 0.0 | 1 | 0.2 | 10th |
| Univ. Alabama | 1 | 0.1 | 0 | 0.0 | 0 | 0.0 | 0 | 0.0 | 0 | 0.0 | 1 | 0.4 | 1 | 0.2 | 10th |
| Univ. Alaska, Fairbanks | 2 | 0.2 | 0 | 0.0 | 0 | 0.0 | 1 | 0.4 | 0 | 0.0 | 1 | 0.4 | 1 | 0.2 | 10th |
| Univ. Arkansas | 1 | 0.1 | 0 | 0.0 | 0 | 0.0 | 0 | 0.0 | 0 | 0.0 | 1 | 0.4 | 1 | 0.2 | 10th |
| Univ. Cincinnati | 1 | 0.1 | 0 | 0.0 | 0 | 0.0 | 0 | 0.0 | 1 | 0.4 | 0 | 0.0 | 1 | 0.2 | 10th |
| Univ. Nebraska, Lincoln | 1 | 0.1 | 0 | 0.0 | 0 | 0.0 | 0 | 0.0 | 0 | 0.0 | 1 | 0.4 | 1 | 0.2 | 10th |
| Univ. Oklahoma | 3 | 0.3 | 0 | 0.0 | 1 | 0.6 | 1 | 0.4 | 0 | 0.0 | 1 | 0.4 | 1 | 0.2 | 10th |
| Univ. South Florida | 1 | 0.1 | 0 | 0.0 | 0 | 0.0 | 0 | 0.0 | 0 | 0.0 | 1 | 0.4 | 1 | 0.2 | 10th |
