# Supplementary material for: Market share and recent hiring trends in anthropology faculty positions
Source: PLoS One. 2018 Sep 12;13(9):e0202528. doi: 10.1371/journal.pone.0202528 (PMC6135356; doi:10.1371/journal.pone.0202528)
Supplement: S3 Table — (DOCX) [file pone.0202528.s003.docx]

**S3 Table. Summary of Biological Anthropology market share divided into 10-year increments (based on when the PhD was awarded, not when they obtained a faculty position) beginning with 1974.** Rankings are based on cumulative market share for the period 1994–2014.

| **University** | **All Years (677)** | | **<1974 (33)** | | **1974-1983 (92)** | | **1984-1993 (116)** | | **1994-2003 (210)** | | **2004-2014 (226)** | | **20 Year Total (436)** | |  |
| --- | --- | --- | --- | --- | --- | --- | --- | --- | --- | --- | --- | --- | --- | --- | --- |
|  | **n** | **%** | **n** | **%** | **n** | **%** | **n** | **%** | **n** | **%** | **n** | **%** | **n** | **%** | **Percentile** |
| Foreign | 33 | 4.8 | 3 | 9.1 | 4 | 4.3 | 3 | 2.5 | 11 | 5.2 | 12 | 5.3 | 23 | 5.3 | 95th |
| SUNY Stony Brook | 26 | 3.8 | 0 | 0.0 | 1 | 1.1 | 5 | 4.2 | 12 | 5.7 | 8 | 3.5 | 20 | 4.6 | 95th |
| Harvard Univ. | 38 | 5.6 | 4 | 12.1 | 3 | 3.2 | 12 | 10.1 | 9 | 4.3 | 10 | 4.4 | 19 | 4.4 | 95th |
| Emory Univ. | 19 | 2.8 | 0 | 0.0 | 0 | 0.0 | 1 | 0.8 | 8 | 3.8 | 10 | 4.4 | 18 | 4.1 | 95th |
| Univ. Michigan, Ann Arbor | 39 | 5.7 | 2 | 6.1 | 9 | 9.7 | 11 | 9.2 | 10 | 4.8 | 7 | 3.1 | 17 | 3.9 | 90th |
| Univ. New Mexico | 18 | 2.6 | 0 | 0.0 | 2 | 2.2 | 1 | 0.8 | 8 | 3.8 | 7 | 3.1 | 15 | 3.4 | 90th |
| Univ. Tennessee, Knoxville | 19 | 2.8 | 0 | 0.0 | 3 | 3.2 | 2 | 1.7 | 8 | 3.8 | 6 | 2.7 | 14 | 3.2 | 90th |
| Ohio St. Univ. | 17 | 2.5 | 0 | 0.0 | 2 | 2.2 | 1 | 0.8 | 5 | 2.4 | 9 | 4.0 | 14 | 3.2 | 90th |
| Univ. California, Davis | 17 | 2.5 | 0 | 0.0 | 4 | 4.3 | 2 | 1.7 | 8 | 3.8 | 3 | 1.3 | 11 | 2.5 | 75th |
| Arizona St. Univ. | 13 | 1.9 | 1 | 3.0 | 0 | 0.0 | 1 | 0.8 | 5 | 2.4 | 6 | 2.7 | 11 | 2.5 | 75th |
| Univ. California, Berkeley | 26 | 3.8 | 4 | 12.1 | 3 | 3.2 | 9 | 7.6 | 7 | 3.3 | 3 | 1.3 | 10 | 2.3 | 75th |
| Pennsylvania St. Univ. | 19 | 2.8 | 2 | 6.1 | 6 | 6.5 | 2 | 1.7 | 4 | 1.9 | 5 | 2.2 | 9 | 2.1 | 75th |
| Yale Univ. | 16 | 2.3 | 1 | 3.0 | 4 | 4.3 | 2 | 1.7 | 6 | 2.9 | 3 | 1.3 | 9 | 2.1 | 75th |
| City Univ. New York | 14 | 2.1 | 1 | 3.0 | 1 | 1.1 | 3 | 2.5 | 5 | 2.4 | 4 | 1.8 | 9 | 2.1 | 75th |
| Indiana Univ., Bloomington | 13 | 1.9 | 0 | 0.0 | 2 | 2.2 | 2 | 1.7 | 4 | 1.9 | 5 | 2.2 | 9 | 2.1 | 75th |
| Washington Univ., St. Louis | 13 | 1.9 | 0 | 0.0 | 1 | 1.1 | 3 | 2.5 | 5 | 2.4 | 4 | 1.8 | 9 | 2.1 | 75th |
| New York Univ. | 12 | 1.8 | 0 | 0.0 | 2 | 2.2 | 1 | 0.8 | 1 | 0.5 | 8 | 3.5 | 9 | 2.1 | 75th |
| Univ. Texas, Austin | 12 | 1.8 | 0 | 0.0 | 2 | 2.2 | 1 | 0.8 | 2 | 1.0 | 7 | 3.1 | 9 | 2.1 | 75th |
| Univ. Chicago | 17 | 2.5 | 2 | 6.1 | 6 | 6.5 | 1 | 0.8 | 6 | 2.9 | 2 | 0.9 | 8 | 1.8 | 75th |
| Univ. California, Los Angeles | 16 | 2.3 | 0 | 0.0 | 4 | 4.3 | 4 | 3.4 | 6 | 2.9 | 2 | 0.9 | 8 | 1.8 | 75th |
| Univ. Colorado, Boulder | 11 | 1.6 | 1 | 3.0 | 1 | 1.1 | 1 | 0.8 | 5 | 2.4 | 3 | 1.3 | 8 | 1.8 | 75th |
| Univ. Florida | 8 | 1.2 | 0 | 0.0 | 0 | 0.0 | 0 | 0.0 | 5 | 2.4 | 3 | 1.3 | 8 | 1.8 | 75th |
| Univ. Pennsylvania | 10 | 1.5 | 0 | 0.0 | 3 | 3.2 | 0 | 0.0 | 3 | 1.4 | 4 | 1.8 | 7 | 1.6 | 50th |
| Univ. California, Santa Barbara | 9 | 1.3 | 0 | 0.0 | 1 | 1.1 | 1 | 0.8 | 4 | 1.9 | 3 | 1.3 | 7 | 1.6 | 50th |
| Tulane Univ. | 7 | 1.0 | 0 | 0.0 | 0 | 0.0 | 0 | 0.0 | 0 | 0.0 | 7 | 3.1 | 7 | 1.6 | 50th |
| Univ. Missouri | 7 | 1.0 | 0 | 0.0 | 0 | 0.0 | 0 | 0.0 | 7 | 3.3 | 0 | 0.0 | 7 | 1.6 | 50th |
| Northwestern Univ. | 13 | 1.9 | 0 | 0.0 | 2 | 2.2 | 5 | 4.2 | 1 | 0.5 | 5 | 2.2 | 6 | 1.4 | 50th |
| Univ. Illinois, Urbana-Champaign | 9 | 1.3 | 2 | 6.1 | 0 | 0.0 | 1 | 0.8 | 3 | 1.4 | 3 | 1.3 | 6 | 1.4 | 50th |
| Rutgers Univ. | 7 | 1.0 | 0 | 0.0 | 1 | 1.1 | 0 | 0.0 | 1 | 0.5 | 5 | 2.2 | 6 | 1.4 | 50th |
| SUNY Binghamton | 7 | 1.0 | 0 | 0.0 | 1 | 1.1 | 0 | 0.0 | 2 | 1.0 | 4 | 1.8 | 6 | 1.4 | 50th |
| Univ. North Carolina, Chapel Hill | 7 | 1.0 | 0 | 0.0 | 0 | 0.0 | 1 | 0.8 | 2 | 1.0 | 4 | 1.8 | 6 | 1.4 | 50th |
| Univ. Arizona | 9 | 1.3 | 0 | 0.0 | 0 | 0.0 | 4 | 3.4 | 2 | 1.0 | 3 | 1.3 | 5 | 1.1 | 50th |
| Columbia Univ. | 7 | 1.0 | 1 | 3.0 | 1 | 1.1 | 0 | 0.0 | 4 | 1.9 | 1 | 0.4 | 5 | 1.1 | 50th |
| SUNY Albany | 6 | 0.9 | 0 | 0.0 | 0 | 0.0 | 1 | 0.8 | 2 | 1.0 | 3 | 1.3 | 5 | 1.1 | 50th |
| Univ. Minnesota | 5 | 0.7 | 0 | 0.0 | 0 | 0.0 | 0 | 0.0 | 3 | 1.4 | 2 | 0.9 | 5 | 1.1 | 50th |
| Univ. Washington | 10 | 1.5 | 1 | 3.0 | 4 | 4.3 | 1 | 0.8 | 1 | 0.5 | 3 | 1.3 | 4 | 0.9 | 50th |
| Duke Univ. | 9 | 1.3 | 1 | 3.0 | 0 | 0.0 | 4 | 3.4 | 1 | 0.5 | 3 | 1.3 | 4 | 0.9 | 50th |
| Univ. Oregon | 8 | 1.2 | 0 | 0.0 | 2 | 2.2 | 2 | 1.7 | 1 | 0.5 | 3 | 1.3 | 4 | 0.9 | 50th |
| Case Western Reserve Univ. | 6 | 0.9 | 0 | 0.0 | 0 | 0.0 | 2 | 1.7 | 4 | 1.9 | 0 | 0.0 | 4 | 0.9 | 50th |
| Kent St. Univ. | 6 | 0.9 | 0 | 0.0 | 0 | 0.0 | 2 | 1.7 | 2 | 1.0 | 2 | 0.9 | 4 | 0.9 | 50th |
| Univ. Pittsburgh | 6 | 0.9 | 0 | 0.0 | 1 | 1.1 | 1 | 0.8 | 2 | 1.0 | 2 | 0.9 | 4 | 0.9 | 50th |
| Stanford Univ. | 5 | 0.7 | 1 | 3.0 | 0 | 0.0 | 0 | 0.0 | 3 | 1.4 | 1 | 0.4 | 4 | 0.9 | 50th |
| Univ. California, Santa Cruz | 4 | 0.6 | 0 | 0.0 | 0 | 0.0 | 0 | 0.0 | 0 | 0.0 | 4 | 1.8 | 4 | 0.9 | 50th |
| Univ. Massachusetts, Amherst | 15 | 2.2 | 2 | 6.1 | 3 | 3.2 | 7 | 5.9 | 1 | 0.5 | 2 | 0.9 | 3 | 0.7 | 25th |
| Johns Hopkins Univ. | 5 | 0.7 | 0 | 0.0 | 0 | 0.0 | 2 | 1.7 | 0 | 0.0 | 3 | 1.3 | 3 | 0.7 | 25th |
| SUNY Buffalo | 5 | 0.7 | 0 | 0.0 | 1 | 1.1 | 1 | 0.8 | 1 | 0.5 | 2 | 0.9 | 3 | 0.7 | 25th |
| Cornell Univ. | 4 | 0.6 | 1 | 3.0 | 0 | 0.0 | 0 | 0.0 | 1 | 0.5 | 2 | 0.9 | 3 | 0.7 | 25th |
| Southern Illinois Univ., Carbondale | 3 | 0.4 | 0 | 0.0 | 0 | 0.0 | 0 | 0.0 | 1 | 0.5 | 2 | 0.9 | 3 | 0.7 | 25th |
| Univ. Arkansas | 3 | 0.4 | 0 | 0.0 | 0 | 0.0 | 0 | 0.0 | 1 | 0.5 | 2 | 0.9 | 3 | 0.7 | 25th |
| Univ. Georgia | 3 | 0.4 | 0 | 0.0 | 0 | 0.0 | 0 | 0.0 | 2 | 1.0 | 1 | 0.4 | 3 | 0.7 | 25th |
| Univ. Nevada, Las Vegas | 3 | 0.4 | 0 | 0.0 | 0 | 0.0 | 0 | 0.0 | 0 | 0.0 | 3 | 1.3 | 3 | 0.7 | 25th |
| Univ. Connecticut | 5 | 0.7 | 0 | 0.0 | 3 | 3.2 | 0 | 0.0 | 1 | 0.5 | 1 | 0.4 | 2 | 0.5 | 25th |
| Washington St. Univ. | 4 | 0.6 | 0 | 0.0 | 1 | 1.1 | 1 | 0.8 | 0 | 0.0 | 2 | 0.9 | 2 | 0.5 | 25th |
| Purdue Univ. | 3 | 0.4 | 0 | 0.0 | 0 | 0.0 | 1 | 0.8 | 1 | 0.5 | 1 | 0.4 | 2 | 0.5 | 25th |
| Temple Univ. | 3 | 0.4 | 0 | 0.0 | 0 | 0.0 | 1 | 0.8 | 0 | 0.0 | 2 | 0.9 | 2 | 0.5 | 25th |
| Univ. California, San Diego | 3 | 0.4 | 0 | 0.0 | 0 | 0.0 | 1 | 0.8 | 1 | 0.5 | 1 | 0.4 | 2 | 0.5 | 25th |
| Princeton Univ. | 2 | 0.3 | 0 | 0.0 | 0 | 0.0 | 0 | 0.0 | 1 | 0.5 | 1 | 0.4 | 2 | 0.5 | 25th |
| Texas A&M Univ. | 2 | 0.3 | 0 | 0.0 | 0 | 0.0 | 0 | 0.0 | 0 | 0.0 | 2 | 0.9 | 2 | 0.5 | 25th |
| Univ. Alabama | 2 | 0.3 | 0 | 0.0 | 0 | 0.0 | 0 | 0.0 | 0 | 0.0 | 2 | 0.9 | 2 | 0.5 | 25th |
| Univ. Illinois, Chicago | 2 | 0.3 | 0 | 0.0 | 0 | 0.0 | 0 | 0.0 | 0 | 0.0 | 2 | 0.9 | 2 | 0.5 | 25th |
| Univ. Kentucky | 2 | 0.3 | 0 | 0.0 | 0 | 0.0 | 0 | 0.0 | 0 | 0.0 | 2 | 0.9 | 2 | 0.5 | 25th |
| Michigan St. Univ. | 6 | 0.9 | 1 | 3.0 | 1 | 1.1 | 3 | 2.5 | 0 | 0.0 | 1 | 0.4 | 1 | 0.2 | 10th |
| Univ. Kansas | 6 | 0.9 | 1 | 3.0 | 1 | 1.1 | 3 | 2.5 | 1 | 0.5 | 0 | 0.0 | 1 | 0.2 | 10th |
| Univ. Utah | 5 | 0.7 | 0 | 0.0 | 3 | 3.2 | 1 | 0.8 | 1 | 0.5 | 0 | 0.0 | 1 | 0.2 | 10th |
| Univ. Wisconsin, Madison | 4 | 0.6 | 1 | 3.0 | 1 | 1.1 | 1 | 0.8 | 1 | 0.5 | 0 | 0.0 | 1 | 0.2 | 10th |
| Syracuse Univ. | 3 | 0.4 | 0 | 0.0 | 2 | 2.2 | 0 | 0.0 | 1 | 0.5 | 0 | 0.0 | 1 | 0.2 | 10th |
| Brown Univ. | 2 | 0.3 | 0 | 0.0 | 0 | 0.0 | 1 | 0.8 | 0 | 0.0 | 1 | 0.4 | 1 | 0.2 | 10th |
| American Univ. | 1 | 0.1 | 0 | 0.0 | 0 | 0.0 | 0 | 0.0 | 0 | 0.0 | 1 | 0.4 | 1 | 0.2 | 10th |
| Florida St. Univ. | 1 | 0.1 | 0 | 0.0 | 0 | 0.0 | 0 | 0.0 | 0 | 0.0 | 1 | 0.4 | 1 | 0.2 | 10th |
| Univ. Alaska, Fairbanks | 1 | 0.1 | 0 | 0.0 | 0 | 0.0 | 0 | 0.0 | 1 | 0.5 | 0 | 0.0 | 1 | 0.2 | 10th |
| Univ. California, Irvine | 1 | 0.1 | 0 | 0.0 | 0 | 0.0 | 0 | 0.0 | 1 | 0.5 | 0 | 0.0 | 1 | 0.2 | 10th |
| Univ. California, Riverside | 1 | 0.1 | 0 | 0.0 | 0 | 0.0 | 0 | 0.0 | 1 | 0.5 | 0 | 0.0 | 1 | 0.2 | 10th |
| Univ. Hawaii | 1 | 0.1 | 0 | 0.0 | 0 | 0.0 | 0 | 0.0 | 1 | 0.5 | 0 | 0.0 | 1 | 0.2 | 10th |
| Univ. Iowa | 1 | 0.1 | 0 | 0.0 | 0 | 0.0 | 0 | 0.0 | 1 | 0.5 | 0 | 0.0 | 1 | 0.2 | 10th |
| Univ. Nevada, Reno | 1 | 0.1 | 0 | 0.0 | 0 | 0.0 | 0 | 0.0 | 1 | 0.5 | 0 | 0.0 | 1 | 0.2 | 10th |
| Univ. South Carolina | 1 | 0.1 | 0 | 0.0 | 0 | 0.0 | 0 | 0.0 | 1 | 0.5 | 0 | 0.0 | 1 | 0.2 | 10th |
