# Supplementary material for: Market share and recent hiring trends in anthropology faculty positions
Source: PLoS One. 2018 Sep 12;13(9):e0202528. doi: 10.1371/journal.pone.0202528 (PMC6135356; doi:10.1371/journal.pone.0202528)
Supplement: S4 Table — (DOCX) [file pone.0202528.s004.docx]

**S4 Table. Summary of Sociocultural Anthropology market share divided into 10-year increments (based on when the PhD was awarded, not when they obtained a faculty position) beginning with 1974.** Rankings are based on cumulative market share for the period 1994–2014.

| **University** | **All Years (1920)** | | **<1974 (91)** | | **1974-1983 (293)** | | **1984-1993 (382)** | | **1994-2003 (588)** | | **2004-2014 (566)** | | **20 Year Total (1154)** | |  |
| --- | --- | --- | --- | --- | --- | --- | --- | --- | --- | --- | --- | --- | --- | --- | --- |
|  | **n** | **%** | **n** | **%** | **n** | **%** | **n** | **%** | **n** | **%** | **n** | **%** | **n** | **%** | **Percentile** |
| Univ. Chicago | 178 | 9.2 | 19 | 20.7 | 28 | 9.4 | 36 | 9.4 | 44 | 7.5 | 51 | 9.0 | 95 | 8.2 | 95th |
| Foreign | 110 | 5.7 | 14 | 15.2 | 15 | 5.1 | 20 | 5.2 | 36 | 6.1 | 25 | 4.4 | 61 | 5.3 | 95th |
| Univ. Michigan, Ann Arbor | 84 | 4.4 | 1 | 1.1 | 13 | 4.4 | 12 | 3.1 | 31 | 5.3 | 27 | 4.8 | 58 | 5.0 | 95th |
| Univ. California, Berkeley | 105 | 5.4 | 6 | 6.5 | 19 | 6.4 | 25 | 6.5 | 31 | 5.3 | 24 | 4.2 | 55 | 4.8 | 95th |
| Harvard Univ. | 85 | 4.4 | 6 | 6.5 | 13 | 4.4 | 24 | 6.3 | 24 | 4.1 | 18 | 3.2 | 42 | 3.6 | 95th |
| New York Univ. | 46 | 2.4 | 1 | 1.1 | 4 | 1.3 | 3 | 0.8 | 14 | 2.4 | 24 | 4.2 | 38 | 3.3 | 90th |
| Univ. Arizona | 45 | 2.3 | 1 | 1.1 | 5 | 1.7 | 5 | 1.3 | 14 | 2.4 | 20 | 3.5 | 34 | 2.9 | 90th |
| Univ. Texas, Austin | 48 | 2.5 | 0 | 0.0 | 5 | 1.7 | 10 | 2.6 | 10 | 1.7 | 23 | 4.1 | 33 | 2.9 | 90th |
| Columbia Univ. | 74 | 3.8 | 8 | 8.7 | 18 | 6.1 | 17 | 4.4 | 11 | 1.9 | 20 | 3.5 | 31 | 2.7 | 90th |
| Stanford Univ. | 65 | 3.4 | 1 | 1.1 | 15 | 5.1 | 18 | 4.7 | 22 | 3.7 | 9 | 1.6 | 31 | 2.7 | 90th |
| Yale Univ. | 41 | 2.1 | 0 | 0.0 | 7 | 2.4 | 4 | 1.0 | 18 | 3.1 | 12 | 2.1 | 30 | 2.6 | 75th |
| Univ. California, Los Angeles | 49 | 2.5 | 1 | 1.1 | 2 | 0.7 | 17 | 4.4 | 16 | 2.7 | 13 | 2.3 | 29 | 2.5 | 75th |
| Univ. Pennsylvania | 47 | 2.4 | 1 | 1.1 | 8 | 2.7 | 10 | 2.6 | 12 | 2.0 | 16 | 2.8 | 28 | 2.4 | 75th |
| City Univ. New York | 45 | 2.3 | 0 | 0.0 | 5 | 1.7 | 14 | 3.6 | 13 | 2.2 | 13 | 2.3 | 26 | 2.3 | 75th |
| Indiana Univ., Bloomington | 45 | 2.3 | 1 | 1.1 | 9 | 3.0 | 10 | 2.6 | 14 | 2.4 | 11 | 1.9 | 25 | 2.2 | 75th |
| Cornell Univ. | 41 | 2.1 | 2 | 2.2 | 6 | 2.0 | 10 | 2.6 | 11 | 1.9 | 12 | 2.1 | 23 | 2.0 | 75th |
| Univ. Illinois, Urbana-Champaign | 39 | 2.0 | 1 | 1.1 | 9 | 3.0 | 7 | 1.8 | 14 | 2.4 | 8 | 1.4 | 22 | 1.9 | 75th |
| Univ. Washington | 29 | 1.5 | 1 | 1.1 | 3 | 1.0 | 4 | 1.0 | 12 | 2.0 | 9 | 1.6 | 21 | 1.8 | 75th |
| Duke Univ. | 22 | 1.1 | 0 | 0.0 | 4 | 1.3 | 1 | 0.3 | 8 | 1.4 | 9 | 1.6 | 17 | 1.5 | 75th |
| Univ. California, San Diego | 32 | 1.7 | 0 | 0.0 | 5 | 1.7 | 10 | 2.6 | 11 | 1.9 | 6 | 1.1 | 17 | 1.5 | 75th |
| Univ. Florida | 27 | 1.4 | 0 | 0.0 | 4 | 1.3 | 6 | 1.6 | 7 | 1.2 | 10 | 1.8 | 17 | 1.5 | 75th |
| Johns Hopkins Univ. | 23 | 1.2 | 0 | 0.0 | 3 | 1.0 | 4 | 1.0 | 12 | 2.0 | 4 | 0.7 | 16 | 1.4 | 75th |
| Univ. California, Santa Cruz | 18 | 0.9 | 0 | 0.0 | 1 | 0.3 | 1 | 0.3 | 7 | 1.2 | 9 | 1.6 | 16 | 1.4 | 75th |
| Univ. Wisconsin, Madison | 29 | 1.5 | 2 | 2.2 | 2 | 0.7 | 9 | 2.3 | 7 | 1.2 | 9 | 1.6 | 16 | 1.4 | 75th |
| Emory Univ. | 14 | 0.7 | 0 | 0.0 | 0 | 0.0 | 0 | 0.0 | 6 | 1.0 | 8 | 1.4 | 14 | 1.2 | 75th |
| Univ. California, Davis | 18 | 0.9 | 0 | 0.0 | 1 | 0.3 | 3 | 0.8 | 7 | 1.2 | 7 | 1.2 | 14 | 1.2 | 75th |
| Univ. Georgia | 15 | 0.8 | 0 | 0.0 | 0 | 0.0 | 1 | 0.3 | 6 | 1.0 | 8 | 1.4 | 14 | 1.2 | 75th |
| Univ. New Mexico | 16 | 0.8 | 0 | 0.0 | 0 | 0.0 | 2 | 0.5 | 8 | 1.4 | 6 | 1.1 | 14 | 1.2 | 75th |
| Univ. Virginia | 19 | 1.0 | 0 | 0.0 | 2 | 0.7 | 3 | 0.8 | 8 | 1.4 | 6 | 1.1 | 14 | 1.2 | 75th |
| Univ. North Carolina, Chapel Hill | 18 | 0.9 | 2 | 2.2 | 3 | 1.0 | 0 | 0.0 | 9 | 1.5 | 4 | 0.7 | 13 | 1.1 | 50th |
| Rutgers Univ. | 15 | 0.8 | 0 | 0.0 | 0 | 0.0 | 3 | 0.8 | 5 | 0.9 | 7 | 1.2 | 12 | 1.0 | 50th |
| Temple Univ. | 17 | 0.9 | 0 | 0.0 | 1 | 0.3 | 4 | 1.0 | 5 | 0.9 | 7 | 1.2 | 12 | 1.0 | 50th |
| Arizona St. Univ. | 12 | 0.6 | 0 | 0.0 | 0 | 0.0 | 1 | 0.3 | 8 | 1.4 | 3 | 0.5 | 11 | 1.0 | 50th |
| Univ. California, Santa Barbara | 22 | 1.1 | 1 | 1.1 | 5 | 1.7 | 5 | 1.3 | 4 | 0.7 | 7 | 1.2 | 11 | 1.0 | 50th |
| Michigan St. Univ. | 13 | 0.7 | 0 | 0.0 | 1 | 0.3 | 2 | 0.5 | 5 | 0.9 | 5 | 0.9 | 10 | 0.9 | 50th |
| Univ. Pittsburgh | 18 | 0.9 | 2 | 2.2 | 4 | 1.3 | 2 | 0.5 | 5 | 0.9 | 5 | 0.9 | 10 | 0.9 | 50th |
| Princeton Univ. | 19 | 1.0 | 0 | 0.0 | 7 | 2.4 | 3 | 0.8 | 6 | 1.0 | 3 | 0.5 | 9 | 0.8 | 50th |
| SUNY Albany | 14 | 0.7 | 0 | 0.0 | 2 | 0.7 | 3 | 0.8 | 4 | 0.7 | 5 | 0.9 | 9 | 0.8 | 50th |
| Univ. California, Irvine | 12 | 0.6 | 0 | 0.0 | 2 | 0.7 | 1 | 0.3 | 3 | 0.5 | 6 | 1.1 | 9 | 0.8 | 50th |
| Washington Univ., St. Louis | 10 | 0.5 | 0 | 0.0 | 1 | 0.3 | 0 | 0.0 | 5 | 0.9 | 4 | 0.7 | 9 | 0.8 | 50th |
| Brown Univ. | 14 | 0.7 | 0 | 0.0 | 3 | 1.0 | 3 | 0.8 | 3 | 0.5 | 5 | 0.9 | 8 | 0.7 | 50th |
| Univ. Oregon | 13 | 0.7 | 3 | 3.3 | 0 | 0.0 | 2 | 0.5 | 4 | 0.7 | 4 | 0.7 | 8 | 0.7 | 50th |
| American Univ. | 15 | 0.8 | 0 | 0.0 | 4 | 1.3 | 4 | 1.0 | 4 | 0.7 | 3 | 0.5 | 7 | 0.6 | 50th |
| Northwestern Univ. | 22 | 1.1 | 4 | 4.3 | 6 | 2.0 | 5 | 1.3 | 3 | 0.5 | 4 | 0.7 | 7 | 0.6 | 50th |
| Pennsylvania St. Univ. | 13 | 0.7 | 0 | 0.0 | 2 | 0.7 | 4 | 1.0 | 3 | 0.5 | 4 | 0.7 | 7 | 0.6 | 50th |
| Tulane Univ. | 7 | 0.4 | 0 | 0.0 | 0 | 0.0 | 0 | 0.0 | 5 | 0.9 | 2 | 0.4 | 7 | 0.6 | 50th |
| Univ. Massachusetts, Amherst | 17 | 0.9 | 0 | 0.0 | 5 | 1.7 | 5 | 1.3 | 5 | 0.9 | 2 | 0.4 | 7 | 0.6 | 50th |
| Univ. Oklahoma | 11 | 0.6 | 0 | 0.0 | 1 | 0.3 | 3 | 0.8 | 7 | 1.2 | 0 | 0.0 | 7 | 0.6 | 50th |
| Univ. Southern California | 8 | 0.4 | 0 | 0.0 | 1 | 0.3 | 0 | 0.0 | 5 | 0.9 | 2 | 0.4 | 7 | 0.6 | 50th |
| New School | 14 | 0.7 | 0 | 0.0 | 3 | 1.0 | 5 | 1.3 | 2 | 0.3 | 4 | 0.7 | 6 | 0.5 | 50th |
| Rice Univ. | 9 | 0.5 | 0 | 0.0 | 1 | 0.3 | 2 | 0.5 | 1 | 0.2 | 5 | 0.9 | 6 | 0.5 | 50th |
| Univ. Hawaii | 8 | 0.4 | 0 | 0.0 | 0 | 0.0 | 2 | 0.5 | 3 | 0.5 | 3 | 0.5 | 6 | 0.5 | 50th |
| Univ. Kentucky | 10 | 0.5 | 1 | 1.1 | 0 | 0.0 | 3 | 0.8 | 1 | 0.2 | 5 | 0.9 | 6 | 0.5 | 50th |
| Washington St. Univ. | 13 | 0.7 | 0 | 0.0 | 4 | 1.3 | 3 | 0.8 | 2 | 0.3 | 4 | 0.7 | 6 | 0.5 | 50th |
| Boston Univ. | 11 | 0.6 | 0 | 0.0 | 4 | 1.3 | 2 | 0.5 | 3 | 0.5 | 2 | 0.4 | 5 | 0.4 | 25th |
| SUNY Buffalo | 10 | 0.5 | 0 | 0.0 | 3 | 1.0 | 2 | 0.5 | 4 | 0.7 | 1 | 0.2 | 5 | 0.4 | 25th |
| Syracuse Univ. | 6 | 0.3 | 1 | 1.1 | 0 | 0.0 | 1 | 0.3 | 3 | 0.5 | 1 | 0.2 | 4 | 0.3 | 25th |
| Univ. Colorado, Boulder | 13 | 0.7 | 1 | 1.1 | 4 | 1.3 | 4 | 1.0 | 1 | 0.2 | 3 | 0.5 | 4 | 0.3 | 25th |
| Univ. Iowa | 7 | 0.4 | 1 | 1.1 | 1 | 0.3 | 1 | 0.3 | 3 | 0.5 | 1 | 0.2 | 4 | 0.3 | 25th |
| Univ. Minnesota | 7 | 0.4 | 1 | 1.1 | 2 | 0.7 | 0 | 0.0 | 1 | 0.2 | 3 | 0.5 | 4 | 0.3 | 25th |
| Brandeis Univ. | 8 | 0.4 | 0 | 0.0 | 3 | 1.0 | 2 | 0.5 | 2 | 0.3 | 1 | 0.2 | 3 | 0.3 | 25th |
| Florida St. Univ. | 3 | 0.2 | 0 | 0.0 | 0 | 0.0 | 0 | 0.0 | 1 | 0.2 | 2 | 0.4 | 3 | 0.3 | 25th |
| Ohio St. Univ. | 5 | 0.3 | 0 | 0.0 | 1 | 0.3 | 1 | 0.3 | 3 | 0.5 | 0 | 0.0 | 3 | 0.3 | 25th |
| Southern Illinois Univ., Carbondale | 4 | 0.2 | 0 | 0.0 | 0 | 0.0 | 1 | 0.3 | 2 | 0.3 | 1 | 0.2 | 3 | 0.3 | 25th |
| Univ. California, Riverside | 6 | 0.3 | 0 | 0.0 | 2 | 0.7 | 1 | 0.3 | 1 | 0.2 | 2 | 0.4 | 3 | 0.3 | 25th |
| Univ. Illinois, Chicago | 3 | 0.2 | 0 | 0.0 | 0 | 0.0 | 0 | 0.0 | 0 | 0.0 | 3 | 0.5 | 3 | 0.3 | 25th |
| Univ. Kansas | 3 | 0.2 | 0 | 0.0 | 0 | 0.0 | 0 | 0.0 | 2 | 0.3 | 1 | 0.2 | 3 | 0.3 | 25th |
| Univ. Maryland | 4 | 0.2 | 1 | 1.1 | 0 | 0.0 | 0 | 0.0 | 0 | 0.0 | 3 | 0.5 | 3 | 0.3 | 25th |
| Univ. Nebraska, Lincoln | 3 | 0.2 | 0 | 0.0 | 0 | 0.0 | 0 | 0.0 | 0 | 0.0 | 3 | 0.5 | 3 | 0.3 | 25th |
| Univ. Rochester | 10 | 0.5 | 4 | 4.3 | 3 | 1.0 | 0 | 0.0 | 3 | 0.5 | 0 | 0.0 | 3 | 0.3 | 25th |
| Univ. South Florida | 3 | 0.2 | 0 | 0.0 | 0 | 0.0 | 0 | 0.0 | 1 | 0.2 | 2 | 0.4 | 3 | 0.3 | 25th |
| Southern Methodist Univ. | 5 | 0.3 | 2 | 2.2 | 0 | 0.0 | 1 | 0.3 | 2 | 0.3 | 0 | 0.0 | 2 | 0.2 | 10th |
| SUNY Binghamton | 8 | 0.4 | 0 | 0.0 | 2 | 0.7 | 4 | 1.0 | 0 | 0.0 | 2 | 0.4 | 2 | 0.2 | 10th |
| Univ. Alabama | 2 | 0.1 | 0 | 0.0 | 0 | 0.0 | 0 | 0.0 | 0 | 0.0 | 2 | 0.4 | 2 | 0.2 | 10th |
| Univ. Connecticut | 3 | 0.2 | 0 | 0.0 | 1 | 0.3 | 0 | 0.0 | 1 | 0.2 | 1 | 0.2 | 2 | 0.2 | 10th |
| Univ. Tennessee, Knoxville | 2 | 0.1 | 0 | 0.0 | 0 | 0.0 | 0 | 0.0 | 1 | 0.2 | 1 | 0.2 | 2 | 0.2 | 10th |
| Boston Coll. | 1 | 0.1 | 0 | 0.0 | 0 | 0.0 | 0 | 0.0 | 0 | 0.0 | 1 | 0.2 | 1 | 0.1 | 10th |
| Case Western Reserve Univ. | 2 | 0.1 | 0 | 0.0 | 1 | 0.3 | 0 | 0.0 | 1 | 0.2 | 0 | 0.0 | 1 | 0.1 | 10th |
| Catholic Univ. America | 3 | 0.2 | 1 | 1.1 | 0 | 0.0 | 1 | 0.3 | 1 | 0.2 | 0 | 0.0 | 1 | 0.1 | 10th |
| Clark Univ. | 2 | 0.1 | 0 | 0.0 | 0 | 0.0 | 1 | 0.3 | 1 | 0.2 | 0 | 0.0 | 1 | 0.1 | 10th |
| Colorado St. Univ. | 1 | 0.1 | 0 | 0.0 | 0 | 0.0 | 0 | 0.0 | 0 | 0.0 | 1 | 0.2 | 1 | 0.1 | 10th |
| Louisiana St. Univ. | 1 | 0.1 | 0 | 0.0 | 0 | 0.0 | 0 | 0.0 | 1 | 0.2 | 0 | 0.0 | 1 | 0.1 | 10th |
| Massachusetts Institute Technology | 3 | 0.2 | 0 | 0.0 | 0 | 0.0 | 2 | 0.5 | 1 | 0.2 | 0 | 0.0 | 1 | 0.1 | 10th |
| Montana St. Univ. | 1 | 0.1 | 0 | 0.0 | 0 | 0.0 | 0 | 0.0 | 1 | 0.2 | 0 | 0.0 | 1 | 0.1 | 10th |
| North Carolina St. Univ. | 2 | 0.1 | 0 | 0.0 | 0 | 0.0 | 1 | 0.3 | 0 | 0.0 | 1 | 0.2 | 1 | 0.1 | 10th |
| Purdue Univ. | 1 | 0.1 | 0 | 0.0 | 0 | 0.0 | 0 | 0.0 | 1 | 0.2 | 0 | 0.0 | 1 | 0.1 | 10th |
| Rensselaer Polytechnic Institute | 1 | 0.1 | 0 | 0.0 | 0 | 0.0 | 0 | 0.0 | 0 | 0.0 | 1 | 0.2 | 1 | 0.1 | 10th |
| SUNY Stony Brook | 6 | 0.3 | 0 | 0.0 | 2 | 0.7 | 3 | 0.8 | 1 | 0.2 | 0 | 0.0 | 1 | 0.1 | 10th |
| Texas A&M Univ. | 1 | 0.1 | 0 | 0.0 | 0 | 0.0 | 0 | 0.0 | 1 | 0.2 | 0 | 0.0 | 1 | 0.1 | 10th |
| Texas St. Univ., San Marcos | 1 | 0.1 | 0 | 0.0 | 0 | 0.0 | 0 | 0.0 | 0 | 0.0 | 1 | 0.2 | 1 | 0.1 | 10th |
| Univ. Arkansas | 1 | 0.1 | 0 | 0.0 | 0 | 0.0 | 0 | 0.0 | 0 | 0.0 | 1 | 0.2 | 1 | 0.1 | 10th |
| Univ. California, San Francisco | 2 | 0.1 | 0 | 0.0 | 0 | 0.0 | 1 | 0.3 | 0 | 0.0 | 1 | 0.2 | 1 | 0.1 | 10th |
| Univ. Delaware | 1 | 0.1 | 0 | 0.0 | 0 | 0.0 | 0 | 0.0 | 1 | 0.2 | 0 | 0.0 | 1 | 0.1 | 10th |
| Univ. South Carolina | 1 | 0.1 | 0 | 0.0 | 0 | 0.0 | 0 | 0.0 | 1 | 0.2 | 0 | 0.0 | 1 | 0.1 | 10th |
| Univ. Texas, San Antonio | 1 | 0.1 | 0 | 0.0 | 0 | 0.0 | 0 | 0.0 | 0 | 0.0 | 1 | 0.2 | 1 | 0.1 | 10th |
| Univ. Utah | 1 | 0.1 | 0 | 0.0 | 0 | 0.0 | 0 | 0.0 | 1 | 0.2 | 0 | 0.0 | 1 | 0.1 | 10th |
| Univ. Wisconsin, Milwaukee | 2 | 0.1 | 0 | 0.0 | 1 | 0.3 | 0 | 0.0 | 0 | 0.0 | 1 | 0.2 | 1 | 0.1 | 10th |
| Vanderbilt Univ. | 1 | 0.1 | 0 | 0.0 | 0 | 0.0 | 0 | 0.0 | 0 | 0.0 | 1 | 0.2 | 1 | 0.1 | 10th |
| Wayne St. Univ. | 4 | 0.2 | 0 | 0.0 | 1 | 0.3 | 2 | 0.5 | 0 | 0.0 | 1 | 0.2 | 1 | 0.1 | 10th |
| Wesleyan | 1 | 0.1 | 0 | 0.0 | 0 | 0.0 | 0 | 0.0 | 1 | 0.2 | 0 | 0.0 | 1 | 0.1 | 10th |
