# Supplementary material for: Market share and recent hiring trends in anthropology faculty positions
Source: PLoS One. 2018 Sep 12;13(9):e0202528. doi: 10.1371/journal.pone.0202528 (PMC6135356; doi:10.1371/journal.pone.0202528)
Supplement: S5 Table — (DOCX) [file pone.0202528.s005.docx]

**S5 Table. Summary statistics for ranking and placement averages by subdiscipline for programs listed in Tables S2-S4.** A: archaeology, B: biological anthropology, C: sociocultural anthropology.

| **A**  Archaeology | | | | | | |
| --- | --- | --- | --- | --- | --- | --- |
| Percentile | Institutions | Placements (1994-2014) | Market Share % (1994-2014) | 20-year placement average per institution | Average placement per institution per year | Average years per placement |
| 95th | 4 | 110 | 22.9 | 27.5 ± 3.5 | 1.38 | 0.7 |
| 90th | 4 | 66 | 13.8 | 16.5 ± 1.3 | 0.83 | 1.2 |
| 75th | 13 | 130 | 27.1 | 10 ± 1.5 | 0.50 | 2.0 |
| 50th | 17 | 102 | 21.3 | 6 ± 0.9 | 0.30 | 3.3 |
| 25th | 11 | 37 | 7.7 | 3.4 ± 0.5 | 0.17 | 5.9 |
| 10th | 26 | 35 | 7.3 | 1.3 ± 0.5 | 0.07 | 14.9 |

| **B** Biological Anthropology | | | | | | |
| --- | --- | --- | --- | --- | --- | --- |
| Percentile | Institutions | Placements (1994-2014) | Market Share % (1994-2014) | 20-year placement average per institution | Average placement per institution per year | Average years per placement |
| 95th | 4 | 80 | 18.3 | 20.0 ± 2.2 | 1.00 | 1.0 |
| 90th | 4 | 60 | 13.8 | 15.0 ± 1.4 | 0.75 | 1.3 |
| 75th | 14 | 127 | 29.1 | 9.1 ± 1 | 0.45 | 2.2 |
| 50th | 21 | 110 | 25.2 | 5.2 ± 1.2 | 0.26 | 3.8 |
| 25th | 18 | 44 | 10.1 | 2.4 ± 0.5 | 0.12 | 8.2 |
| 10th | 15 | 15 | 3.4 | 1 | 0.05 | 20.0 |

| **C** Sociocultural Anthropology | | | | | | |
| --- | --- | --- | --- | --- | --- | --- |
| Percentile | Institutions | Placements (1994-2014) | Market Share % (1994-2014) | 20-year placement average per institution | Average placement per institution per year | Average years per placement |
| 95th | 5 | 311 | 26.9 | 62.2 ± 19.7 | 3.11 | 0.3 |
| 90th | 5 | 167 | 14.5 | 33.4 ± 2.9 | 1.67 | 0.6 |
| 75th | 19 | 373 | 32.3 | 19.6 ± 5.6 | 0.98 | 1.0 |
| 50th | 25 | 210 | 18.2 | 8.4 ± 2.1 | 0.42 | 2.4 |
| 25th | 17 | 59 | 5.1 | 3.5 ± 0.7 | 0.17 | 5.8 |
| 10th | 29 | 34 | 2.9 | 1.2 ± 0.4 | 0.06 | 17.1 |
