# Supplementary material for: Market share and recent hiring trends in anthropology faculty positions
Source: PLoS One. 2018 Sep 12;13(9):e0202528. doi: 10.1371/journal.pone.0202528 (PMC6135356; doi:10.1371/journal.pone.0202528)
Supplement: S6 Table — (DOCX) [file pone.0202528.s006.docx]

**S6 Table. US anthropology doctoral recipients by year relative to numbers of US anthropology doctorates successful in obtaining anthropology faculty positions.** F = female; M = male; Tot. = Total.

|  | **Number of Anthropology PhDs Awarded*** | | | **Numbers of Individuals Successful in Obtaining an Academic Position in Anthropology (by year of graduation)** | | | | | | | | | | | |
| --- | --- | --- | --- | --- | --- | --- | --- | --- | --- | --- | --- | --- | --- | --- | --- |
|  |  |  |  | **Archaeology** | | | **Biological** | | | **Sociocultural** | | | **All Subdisciplines** | | |
| **Year** | **F** | **M** | **Tot.** | **F** | **M** | **Tot.** | **F** | **M** | **Tot.** | **F** | **M** | **Tot.** | **F** | **M** | **Tot.** |
| 1985 | 181 | 172 | 353 | 5 | 16 | 21 | 6 | 6 | 12 | 23 | 14 | 37 | 34 | 36 | 70 |
| 1986 | 197 | 184 | 381 | 10 | 12 | 22 | 6 | 7 | 13 | 21 | 18 | 39 | 37 | 37 | 74 |
| 1987 | 181 | 171 | 352 | 8 | 17 | 25 | 3 | 8 | 11 | 17 | 25 | 42 | 28 | 50 | 78 |
| 1988 | 170 | 155 | 325 | 11 | 16 | 27 | 2 | 6 | 8 | 23 | 16 | 39 | 36 | 38 | 74 |
| 1989 | 148 | 177 | 325 | 7 | 17 | 24 | 5 | 6 | 11 | 16 | 22 | 38 | 28 | 45 | 73 |
| 1990 | 173 | 151 | 324 | 12 | 18 | 30 | 12 | 5 | 17 | 26 | 22 | 48 | 50 | 45 | 95 |
| 1991 | 210 | 131 | 341 | 8 | 16 | 24 | 6 | 6 | 12 | 10 | 17 | 27 | 24 | 39 | 63 |
| 1992 | 155 | 165 | 320 | 5 | 9 | 14 | 8 | 7 | 15 | 19 | 22 | 41 | 32 | 38 | 70 |
| 1993 | 213 | 129 | 342 | 13 | 9 | 22 | 4 | 4 | 8 | 24 | 16 | 40 | 41 | 29 | 70 |
| 1994 | 208 | 176 | 384 | 4 | 13 | 17 | 11 | 3 | 14 | 28 | 21 | 49 | 43 | 37 | 80 |
| 1995 | 219 | 156 | 375 | 6 | 17 | 23 | 6 | 6 | 12 | 33 | 20 | 53 | 45 | 43 | 88 |
| 1996 | 214 | 183 | 397 | 10 | 17 | 27 | 11 | 6 | 17 | 27 | 24 | 51 | 48 | 47 | 95 |
| 1997 | 241 | 193 | 434 | 11 | 13 | 24 | 6 | 9 | 15 | 35 | 24 | 59 | 52 | 46 | 98 |
| 1998 | 239 | 186 | 425 | 11 | 12 | 23 | 14 | 7 | 21 | 24 | 33 | 57 | 49 | 52 | 101 |
| 1999 | 263 | 200 | 463 | 8 | 10 | 18 | 15 | 11 | 26 | 39 | 30 | 69 | 62 | 51 | 113 |
| 2000 | 252 | 194 | 446 | 6 | 14 | 20 | 7 | 9 | 16 | 27 | 25 | 52 | 40 | 48 | 88 |
| 2001 | 244 | 166 | 410 | 7 | 17 | 24 | 14 | 20 | 34 | 45 | 22 | 67 | 66 | 59 | 125 |
| 2002 | 289 | 206 | 495 | 12 | 21 | 33 | 15 | 11 | 26 | 38 | 21 | 59 | 65 | 53 | 118 |
| 2003 | 288 | 184 | 472 | 18 | 20 | 38 | 13 | 16 | 29 | 38 | 34 | 72 | 69 | 70 | 139 |
| 2004 | 293 | 238 | 531 | 10 | 15 | 25 | 18 | 14 | 32 | 37 | 35 | 72 | 65 | 64 | 129 |
| 2005 | 256 | 200 | 456 | 18 | 16 | 34 | 16 | 9 | 25 | 37 | 22 | 59 | 71 | 47 | 118 |
| 2006 | 269 | 203 | 472 | 11 | 18 | 29 | 23 | 16 | 39 | 46 | 33 | 79 | 80 | 67 | 147 |
| 2007 | 316 | 196 | 512 | 12 | 16 | 28 | 12 | 9 | 21 | 32 | 25 | 57 | 56 | 50 | 106 |
| 2008 | 294 | 189 | 483 | 9 | 18 | 27 | 16 | 7 | 23 | 42 | 24 | 66 | 67 | 49 | 116 |
| 2009 | 308 | 195 | 503 | 10 | 14 | 24 | 11 | 6 | 17 | 45 | 18 | 63 | 66 | 38 | 104 |
| 2010 | 306 | 201 | 507 | 8 | 12 | 20 | 10 | 9 | 19 | 28 | 22 | 50 | 46 | 43 | 89 |
| 2011 | 330 | 223 | 553 | 11 | 6 | 17 | 14 | 6 | 20 | 21 | 25 | 46 | 46 | 37 | 83 |
| 2012 | 360 | 187 | 547 | 6 | 10 | 16 | 11 | 2 | 13 | 29 | 17 | 46 | 46 | 29 | 75 |
| 2013 | 362 | 188 | 550 | 3 | 2 | 5 | 7 | 4 | 11 | 10 | 8 | 18 | 20 | 14 | 34 |
| 2014 | 330 | 197 | 527 | 4 | 4 | 8 | 4 | 2 | 6 | 5 | 4 | 9 | 13 | 10 | 23 |
| **Total** | **7509** | **5496** | **13005** | **274** | **415** | **689** | **306** | **237** | **543** | **845** | **659** | **1504** | **1425** | **1311** | **2736** |
| * Only includes individuals who self-reported gender | | | | | | | | | | | | | | | |
